# Young children spontaneously invent three different types of associative tool use behaviour

**DOI:** 10.1017/ehs.2022.4

**Published:** 2022-02-02

**Authors:** E. Reindl, C. Tennie, I. A. Apperly, Z. Lugosi, S. R. Beck

**Affiliations:** 1 School of Psychology, University of Birmingham, Brimingham, UK; 2 Department of Anthropology, Durham University, Durham, UK; 3 School of Psychology and Neuroscience, University of St Andrews, St Andrews, UK; 4 Department for Early Prehistory and Quaternary Ecology, University of Tübingen, Tübingen, Germany; 5 Division of Psychology, University of Stirling, Stirling, UK

**Keywords:** Associative tool use, Metatool use, Sequential tool use, Tool set use, Multifunctional tool use, Tool use, Problem-solving

## Abstract

Associative Tool Use (ATU) describes the use of two or more tools in combination, with the literature further differentiating between Tool set use, Tool composite use, Sequential tool use and Secondary tool use. Research investigating the cognitive processes underlying ATU has shown that some primate and bird species spontaneously invent Tool set and Sequential tool use. Yet studies with humans are sparse. Whether children are also able to spontaneously invent ATU behaviours and at what age this ability emerges is poorly understood. We addressed this gap in the literature with two experiments involving preschoolers (E1, *N* = 66, 3 years 6 months to 4 years 9 months; E2, *N* = 119, 3 years 0 months to 6 years 10 months) who were administered novel tasks measuring Tool set, Metatool and Sequential tool use. Participants needed to solve the tasks individually, without the opportunity for social learning (except for enhancement effects). Children from 3 years of age spontaneously invented all of the types of investigated ATU behaviours. Success rates were low, suggesting that individual invention of ATU in novel tasks is still challenging for preschoolers. We discuss how future studies can use and expand our tasks to deepen our understanding of tool use and problem-solving in humans and non-human animals.

## Graphical abstract

**Figure:**
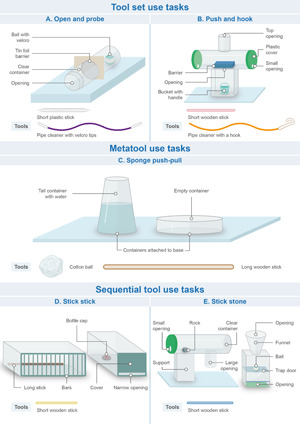

**Social media summary:** Children solve tool-use tasks involving the use of two tools in combination on their own, without social learning.

Tools and technology play a dominant role in the lives of humans of all cultures. They have contributed substantially to the success of our species: our capacities for using, making and innovating tools have opened up new ecological niches (and are still doing so) and contributed to the coevolution of cumulative culture, social learning and teaching (Henrich, 2015; van Schaik et al., 1999). Today, the use and making of many tools has become so complex and/or opaque that they rely on copying of sufficient fidelity, often over extended periods (Gurven et al., 2006; Kaplan & Robson, 2002). Owing to this special role of social learning for the acquisition of many forms of tool use, researchers have investigated when, how and from whom humans, and especially children and adolescents, learn (Bjorklund & Gardiner, 2012; Esseily et al., 2016; Greif & Needham, 2011; Lancy, 2016, 2017; Lew-Levy et al., 2017, 2020; Nagell et al., 1993; Nielsen et al., 2012; Somogyi et al., 2015). These socially learned behaviours add to a phylogenetic baseline of tool use, i.e. a range of behaviours which members of the species in question can acquire *without* the need for copying – if they are at the right environmental, developmental and motivational stage. The current paper explores the contents of this baseline, also called the *Zone of Latent Solutions* (ZLS; Reindl et al., 2018; Tennie et al., 2009, 2020), in humans.

Some of humans’ closest living relatives – chimpanzees and orangutans – also possess varied tool cultures (van Schaik et al., 2003, 2009; Whiten et al., 1999, 2001). However, in contrast to much of human tool culture, there is little evidence that the behaviours of non-human great apes (henceforth apes) require copying know-how (Bandini & Tennie, 2020; Buskell & Tennie, accepted; Motes-Rodrigo & Tennie, 2021; Reindl et al., 2018). Instead, they appear to be *socially mediated reinnovations* (Bandini & Tennie, 2017): while non-copying types of social learning (e.g. local or stimulus enhancement) can guide the individual's attention towards relevant materials and/or locations, each individual re-innovates the form of the behaviour – the know-how – on their own from their ZLS. Owing to their limited engagement in form copying, apes have been suggested to be restricted to ZLS-only cultures. If true, all behavioural forms observed in wild apes are latent solutions (Reindl et al., 2018), and strong evidence for this was provided recently (Motes-Rodrigo & Tennie, 2021). Modern humans – through their capacity for copying know-how – have managed to go beyond their ZLS, i.e. are able to learn and produce culture-dependent forms (i.e. forms which are too complex or arbitrary to be re-innovated from scratch).

By definition, cumulative culture does not arise from zero. The seemingly unbounded landscape of tools and technology modern humans have explored because of their cumulative cultural abilities rests on a second landscape which can be explored by individual learning alone: the ZLS. This second ‘base’ landscape is less well understood (Neldner et al., 2020; Reindl et al., 2016), but can give us important insights – via cognitive cladistics – into the tool-using abilities of our ancestors before the emergence of cumulative culture (Tennie et al., 2016). One way to investigate this landscape is through *latent solution (or baseline) tests*. These are experimental studies in which individuals naive to a cultural behaviour in question are provided with the raw materials necessary to produce the behaviour to study whether they spontaneously re-innovate the behaviour (Bandini & Tennie, 2017; Tennie et al., 2009). While Luria and Vygotsky (1930) thought that all of children's tool use stems from copying and teaching, with children's spontaneous tool use being ‘practically zero’ (p. 114), we now know that infants from 18 months of age can spontaneously use a tool to obtain an out-of-reach object (Fagard et al., 2014; Rat-Fischer et al., 2012) and that from at least 3 years of age children have the capacity to invent the correct solution to simple, but novel, stick tool-use problems on their own, without any immediate help from others (Neldner et al., 2020; Reindl et al., 2016). This might not be surprising, given that the ingredients for successful tool use – increasing motor skills, knowledge about object affordances, refined perception–action routines, causal cognition – are built up gradually from early infancy through social and asocial object exploration and play (Bjorklund & Gardiner, 2012; Chen et al., 2000; Greif & Needham, 2011; Kahrs et al., 2013; Lockman, 2000; Somogyi et al., 2015).

The current study aimed to add to the exploration of the contents of the human ZLS (here by studying a *WEIRD* (Westernized, Educated, Industrialised, Rich, Democratic) population (Henrich et al., 2010), but cross-cultural work is to follow) by asking whether a more complex type of tool use also lies within the human ZLS: namely *Associative Tool Use* (ATU) – the use of two or more tools in combination to achieve a goal (Shumaker et al., 2011). ATU is arguably cognitively more demanding than ‘simple’ tool use (i.e. the use of a single tool; Boesch, 2013): by definition, ATU involves a larger number of objects, and often consists of an increased temporal and/or spatial problem–solution distance, resulting in an increase in the processing complexity of the task and subsequently its cognitive load on working memory and other executive functions such as planning, inhibition, behavioural sequencing and decision making (Haidle, 2010; Halford et al., 1998; Hunt et al., 2013; Read, 2008). Note, however, that the adjective ‘associative’ in ATU should only be understood in its literal meaning (‘relating to, connecting’, from Latin *associatus* (= joined with)), used to describe a type of tool use in which two or more tools are used in combination. It should not be understood to imply a relationship to associative learning (nor any other learning mechanism). In the terminology by Shumaker et al. (2011), simple tool use refers to the use of a single tool, while ATU refers to the use of two or more tools in combination. The question of underlying learning mechanisms is a separate one and not alluded to by using these terms. While simple tool use is assumed to be cognitively less demanding than ATU, this does not imply that simple tool use behaviours can always be acquired by associative learning only.

ATU can be split into several categories, according to the different ways in which tools can be used in combination. To date, there is no uniform nomenclature to describe these different forms (compare e.g. Colbourne et al., 2021; Shumaker et al., 2011; Taylor et al., 2007; Wimpenny et al., 2009). We do not aim to add to the potentially confusing terminology by introducing yet another set of definitions, and therefore refer to the nomenclature used by Shumaker et al. (2011) (Figure 1, Table 1), who have become a widely cited source in the field. We do acknowledge the potentially confusing use of the word ‘associative’, which many comparative psychologists are more familiar with in the context of associative learning, but hope that the above has made clear that the two concepts are not related.

**Figure 1.**
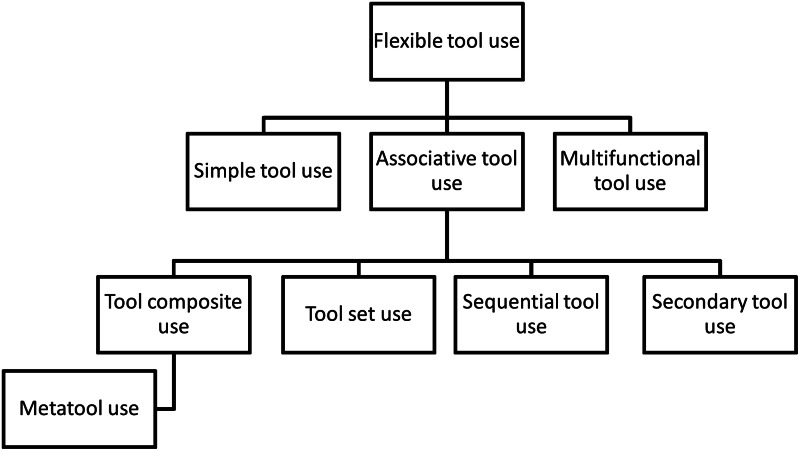
Classification of flexible tool use types as used in this study, based on the definitions in Shumaker, Walkup, and B. B. Beck (2011).

**Table 1.** Associative tool use (ATU) types and their definitions according to Shumaker, Walkup, and B. B. Beck (2011) and list of animals for whom evidence for spontaneous occurrence of ATU exists

ATU type	Definition	Examples (human and non-human)	Non-human animals for which ATU type has been observed to be produced spontaneously (in the wild or in captivity)^a^
Tool set use	Two or more tools used sequentially, usually each in a different mode, to achieve a single outcome	Using a *can opener* to open a tin can followed by scooping the contents with a *spoon*. Using a *stout stick* to open up a termite mound followed by using a *thin twig* to fish for termites	Capuchin monkeys, chimpanzees, Goffin's cockatoos
Tool composite use	Two or more tools used simultaneously, usually each in a different mode, to achieve a single outcome, where the first tool is not used to manufacture the second	Using a *fork* and knife together. Standing on a *box* and using a *stick* to reach a reward	Bonobos, capuchin monkeys, chimpanzees, gorillas, long-tailed macaques, orangutans, satin bowerbirds, sea otters
Metatool use	A tool used simultaneously with a second tool to increase the efficiency of the second tool, where the first tool (metatool) acts directly on the second. The second tool could function as a tool on its own; the metatool makes it a better tool. Every metatool is a tool composite	Using *snow chains* on *car tyres* in winter. Using a *wedge* to stabilize an *anvil*	Capuchin monkeys, chimpanzees, orangutans
Sequential tool use	A tool used to acquire another tool	Using a *ruler* to reach a *key* that has fallen behind the radiator to open a box. Using a *stick* to retrieve a *longer stick* from a box to retrieve a reward from another box	Bonobos, capuchin monkeys, chimpanzees, gorillas, New Caledonian crows, Olive baboons, orangutans, rhesus macaques, rooks
Secondary tool use	A tool used to manufacture (structurally modify) another tool.	Using a *cutting machine* to produce matchsticks. Using a *stone hammer* to produce a *flake for cutting*	Capuchin monkeys

a Based on references in Shumaker et al. (2011) and a literature search for more recent papers, see Table S1 in the OSF repository.

We focused on three out of the four ATU types by Shumaker et al. (2011; for definitions and examples see Table 1): *Tool set use*, *Sequential tool use*, and a special case of using tool composites, *Metatool use.* We did not include Secondary tool use as previous research had shown that tool making and innovation *per se* are difficult for children (Beck et al., 2011; Nielsen et al., 2014; but see Voigt et al., 2019).

ATU has been reported in many species in which flexible (i.e. non-stereotyped) tool use has been observed (for a review, see Table S1 in the OSF repository, which is based on Shumaker et al., 2011, but also comprises research published since then). For example, Tool sets are used by wild chimpanzees and capuchin monkeys: chimpanzees use sets of two or more sticks to get access to beehives, termite mounds or ant nests – stout branches are used as pounding tools and levers to make and widen holes, and finer twigs are used on these holes as dipping sticks (Bernstein-Kurtycz et al., 2020; Shumaker et al., 2011). Capuchin monkeys use stones to pound on beehives or next to cavities, and then use sticks in these holes to probe for honey or small animals (Mannu & Ottoni, 2009). Evidence from latent solution tests with captive chimpanzees (Bernstein-Kurtycz et al., 2020), capuchin monkeys (Westergaard et al., 1997) and Goffin's cockatoos (O'Hara et al., 2021) shows that Tool set use falls into the ZLS of these species.

Metatool use is shown by wild chimpanzees in the context of nut-cracking: when stones are used as hammers and anvils, some individuals at Bossou (Republic of Guinea) use smaller stones as wedges to stabilise the anvils (Carvalho et al., 2008; Matsuzawa, 1991). In addition, wild chimpanzees and captive orangutans have been observed to use a stick to push a leaf/paper into a tree-hole or a puddle of liquid to retrieve water/juice more efficiently (Matsuzawa, 1991, cited in Sugiyama, 1997; Lehner et al., 2011; Lethmate, 1982). Captive capuchin monkeys pounded chisel stones with hammer stones to break open lids, thus being more effective than when using the chisel stones alone (Westergaard et al., 1996; Westergaard & Suomi, 1994a, b). Again, the studies on captive, target behaviour naive individuals suggest that Metatool use is within the ZLS of chimpanzees and capuchin monkeys, i.e. does not need to be copied by others to be acquired.

There are no reports of Sequential tool use in wild non-human animals. Instead, Sequential tool use problems have been created as novel laboratory tasks to investigate problem-solving and causal cognition in various species and date back to Köhler's (1921) work with chimpanzees. Research has shown that New Caledonian crows, all four species of great ape, capuchin monkeys and baboons are able to spontaneously and unaidedly solve Sequential tool use tasks, in which short sticks need to be used to retrieve longer sticks, which in turn can be used to access a reward (Anderson & Henneman, 1994; Bolwig, 1963; Martin-Ordas et al., 2012; Mulcahy et al., 2005; Taylor et al., 2007, 2010; Wimpenny et al., 2009).

In contrast to the non-human animal literature, the low number of studies on ATU in humans is striking. Köhler (1921) remarked that researchers were still facing terra incognita regarding understanding children's flexible tool behaviors, and 90 years later, researchers still pointed out the only patchy understanding of the development of tool use in children (Greif & Needham, 2011). While in the meantime a number of studies on the origins and development of simple tool use, tool making and tool innovation in children have been conducted (Barrett et al., 2007; E. Bates et al., 1980; Bechtel et al., 2013; S. R. Beck et al., 2016; Breyel & Pauen, 2021; Brown, 1990; Chappell et al., 2013; Chen et al., 2000; Deák, 2014; Gönül et al., 2018; Keen, 2010; Lew-Levy et al., 2021; McCarty et al., 1999, 2001; Neldner et al., 2020; Pauen & Bechtel-Kuehne, 2016; Piaget, 1952; Rat-Fischer et al., 2012; Rawlings et al., 2021; Reindl et al., 2016; Voigt et al., 2019; Willatts, 1984), there are only few studies on *associative* tool use in children, and all of them focus on only one type: Sequential tool use (Alpert, 1928; Matheson, 1931; Metevier, 2006). Köhler (1921) remarked that most tool-use behaviours in adults have become ‘mechanised’, i.e. are carried out with ease and so questions about their ontogenetic and phylogenetic origins might not present themselves as very salient. Yet research on ATU allows us to (a) further illuminate the extent of the human ZLS, (b) improve our understanding of the evolution and development of tool-using and innovative skills and their underlying cognitive processes (using cognitive cladistics) and (c) provide insightful comparisons to the existing studies involving ATU in non-humans.

Alpert (1928) and Matheson (1931) adapted Köhler's (1921) tool-use tasks for use with preschool children. In their studies, children were separated from a reward by a railing set up in the testing room. To obtain the reward, children had to use a stick lying on their side of the railing to rake in a longer out-of-reach stick beyond the railing, which they could then use to fetch the reward. Children below 2.5 years of age did not solve this task; only 10 out of the 28 tested 2.5- to 4.5-year-olds (36%) spontaneously solved it (Matheson, 1931). In a more recent study, 3-year-olds completed two tasks involving the use of a single tool before attempting an ATU task (Metevier, 2006): In the ‘tube task’, participants had to use a stick to push a toy out of a tube. In the ‘rake task’, they had to use a rake to obtain an out-of-reach toy on the table. Children found these tasks rather easy, indicated by success rates above 75%. In the ‘combination task’ (ATU), children first had to use the rake to obtain an out-of-reach stick on the table, and subsequently use this stick to push a toy out of the tube. Success rates were low (25–37%), indicating that 3-year-olds struggled with Sequential tool use even though they readily solved the individual components beforehand. These studies suggest that Sequential tool use is challenging for children up to at least the age of 4 years, both when having to solve such tasks spontaneously and given previous experience with individual elements of the tasks. However, no research seems to have been carried out yet on Metatool and Tool set use abilities in children.

The current study aimed to investigate whether young children would be able to solve three types of ATU tasks (Tool set use, Sequential tool use and Metatool use) on their own in a latent solution test, i.e. without immediate social learning (Experiment 1). In Experiment 2 we further compared the difficulty of Tool set and Sequential Tool use using apparatuses that were more comparable between these ATU types. The age range of our samples was slightly higher than in a previous latent solution test on simple tool use (Reindl et al., 2016) as it was assumed that ATU would pose greater demands on executive functions, such as working memory, which are still developing during childhood (Garon et al., 2008). However, note that increasing the age range also increases the amount of previous cultural knowledge that children bring to the experiment and thus the probability that successful children can use previously acquired cultural knowledge to solve the tasks.

## Experiment 1

We investigated whether 3.5- to 4-year-old children would be able to spontaneously solve three types of ATU tasks: Tool set use, Metatool use and Sequential tool use. We created six tasks (two tasks per ATU type); four were based on ATU behaviours observed in wild or captive animals (one Tool set use task, both Metatool use tasks, one Sequential tool use task; see below) and two were new creations. Each child was administered three tasks in a single session, with one task from each ATU category (task order counterbalanced). During data collection, we noticed that one of our Metatool use tasks (*Anvil prop*) had a design flaw as most children (eventually 71%) were able to solve the task in a way not intended by the task design, i.e., without ATU. This task is described in the Supplementary Material but is not included in the analysis.

### Methods

#### Participants

We tested 66 children (31 boys) between 3 years 6 months and 4 years 9 months (dates of birth were known for 64 children: mean ± SD, 4 years 1 month ± 3.88 months) in seven nurseries and a Science museum in Birmingham, UK, between March and July 2014. No *a priori* sample size calculation was carried out for either Experiment 1 or 2; instead, the goal was to test as many children as possible during the time window available for this project. The ethnic composition of the sample was 65.2% White, 21.2% Black and 13.6% Asian. Participants were recruited through letters sent to parents (for children tested in nurseries) and via advertisements on the museum website and social media (for children tested at the museum). Ethical approval for both experiments was granted by the University of Birmingham, UK, STEM Ethical Review Committee.

#### Materials

**Figure 2.**
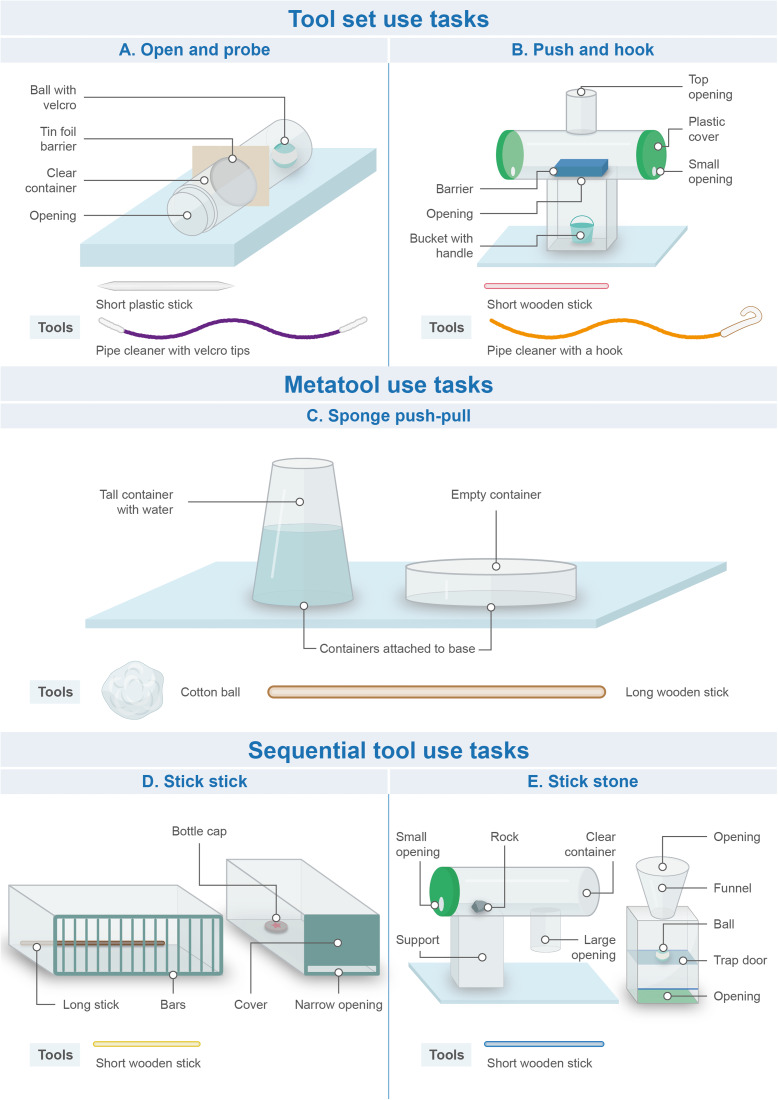
Materials used in Experiment 1. *Note*: Anil prop task not shown as it was excluded from the analysis due to design failure. Figure drawn by Nuria Melisa Morales García.

Drawings of the tasks are displayed in Figure 2 (for photos of the tasks, see Figure S1). For space reasons, tasks are only briefly described here; a full description including animations on how each task could be solved can be found in the Supplementary Material. For each task, the apparatus(es) were positioned in front of the child and all freely accessible tools were placed between the task and the child. Rewards were either stickers placed in containers or other target objects which could be exchanged for a sticker after completion of the task.

##### Tool set use – Open and probe

This task was based on the use of Tool sets by chimpanzees to open beehives, ant nests or termite mounds (see above). The goal was to insert a short, sturdy stick into a tube and pierce a tin foil barrier, and then to use a long pipecleaner with hook and loop fastener wrapped around both ends to reach through the hole in the barrier and retrieve a target object from the back end of the tube.

##### Tool set use – Push and hook

This task was novel. The goal was to insert a short, sturdy stick into the side or top openings of a T-shaped apparatus to move a barrier inside the apparatus, and then to insert a rope with a hook at its end through the top of the apparatus to fish for a bucket containing a reward (this second step was similar to the hook task in Chappell et al., 2013).

##### Metatool use – Sponge push–pull

This task was based on observations of chimpanzees and orangutans using a stick (metatool) to push a leaf/paper towel into a tree-hole or puddle of liquid to retrieve water/juice more efficiently (Matsuzawa, 1991, cited in Sugiyama, 1997; Lehner et al., 2011; Lethmate, 1982). Children were presented with a transparent tube filled with 500 ml of water and a smaller container placed next to it. The goal was to fill the small container with water. Available tools were a wooden stick and a ball of cotton wool. While the stick could be used on its own to solve the task – by dipping it into the tube repeatedly to extract water, this method was inefficient. Instead, the stick use could be improved by the wool as a metatool: children could first drop the wool into the tube so that it could absorb water, after which they could use the stick to retrieve the wool. Note that because of this retrieval action (stick used to retrieve wool from the bottle), the Sponge push–pull task might resemble a Sequential tool use task, which by definition involves the use of a tool to retrieve another tool. However, the retrieval action is just a feature of this particular task. In Metatool use, a tool (here the stick) is used to carry out a target action (here to retrieve the water from the bottle) and the metatool (here the wool) enhances the efficiency of the target action. In contrast, the defining feature of Sequential tool use is that a tool is used to retrieve a second tool in a first step, and then this second tool can be used in a second step to carry out the target action. More information about this differentiation can be found in the Supplementary Material.

##### Sequential tool use – Stick stick

This task was based on an apparatus used for studying Sequential tool use in New Caledonian crows (see figure 1 in Taylor et al., 2007). The goal was to insert a short stick into a box to rake in a longer stick, which could then be used on another box to obtain an-out-of-reach sticker.

##### Sequential tool use – Stick stone

At the time we designed this experiment, this task was novel. Note, however, that a similar design has subsequently been used with New Caledonian crows (Gruber et al., 2019). Metevier (2006) remarked that in most Sequential tool use studies the tools were of the same type (usually sticks of different sizes) and used in a similar fashion (e.g. raking). Therefore, we created a task in which two different kinds of tools (a stick and a stone) had to be used in different ways (pushing, dropping). The goal was to insert a short stick into an apparatus to push out a small stone. The stone could then be dropped into the top of a second apparatus where it would activate a trapdoor and release a target object.

#### Design and procedure

For reasons of practicability, we tested children only once and thus only administered a subset of the tasks (a warm-up game and three tasks) to each child. The combination and order of the tasks were counterbalanced. Across children, every task was presented 24 times, occurring eight times in each of the three positions. Participants were tested individually by the same female experimenter (E.R.) and were sitting at a table or on the floor, perpendicular to the experimenter. A warm-up game was used to familiarise children with breaking and modifying material within the experimental session. This was important as the Anvil prop task required children to apply physical force and break the plastic nut. For the test phase, children were presented with three semi-randomly chosen tasks, one from each ATU category. Tasks were presented as a game to the children in which they could win stickers. Materials were placed in front of the participant, with the tools lying between the apparatus(es) and the child. Children were told the goal of the task, e.g. ‘to get this orange ball out of the bottle’ (Open and probe) and that they could ‘use anything here on the table/floor’ to solve the task, but they were never told that they had to use the tools to solve the tasks and they only received general encouragement. Children had 3 min to solve each game. Trials ended when children obtained the target, when time was over or if children refused to play. When one trial ended, the experimenter cleared the table/floor and fetched the next task. Children were rewarded with stickers regardless of success. Children were never shown the correct solution of a task if they were unsuccessful. Total testing time was ~15 min.

#### Scoring and analysis

Children's behaviour was live-coded and coding was double-checked offline by E.R. using videos from nine children (14% of the sample; note that videos were not available for all participants). For each task, we scored whether children picked up the tool(s), used the tool(s) in the manner intended by the experimenter, engaged in ATU (i.e. whether children used both tools to solve the task in a manner that was intended by us) and whether they solved the task following ATU (*Correct success*; i.e. whether children succeeded after having used both tools in the way intended by us). We scored *Incorrect success* whenever children solved the task in a way that was not intended by us. To obtain inter-observer reliability, 31% of the valid trials (i.e. 50 trials) were live-coded by a second rater who was present during the experiment and asked to code our two main variables, ATU and Correct success. Inter-rater agreement for both variables was perfect (Cohen's *k* = 1.000).

Each of the 66 children participated in three tasks, resulting in 198 trials. From these, we excluded all of the Anvil prop trials (*n* = 33) and a further three trials owing to experimenter error (Tube task, *n* = 2) or because the child became upset (Tube task, *n* = 1), resulting in a final number of 162 valid trials across five tasks.

Analyses were carried out in R version 4.0.3 (R Core Team, 2020). To address our main question – whether children were able to spontaneously engage in ATU to solve novel problems – we carried out descriptive analyses for each task, investigating success rates. In exploratory analyses, we examined whether the two tasks within the Tool set and Sequential tool use types were of comparable difficulty by conducting chi-square analyses of children's rates of ATU and Correct success. We also examined whether ATU type affected children's ATU and Correct success using two Generalised Linear Mixed Models (GLMMs; Baayen, 2008) with binomial error structure and logit link function (McCullagh & Nelder, 1989) implemented by the *glmer* function of the R package *lme4* (D. M. Bates et al., 2013). In both models, age in months (*z*-transformed to a mean of zero and a standard deviation of 1) was entered as control variable and ATU type as the predictor variable. Participant ID was included as a random effect to account for the fact that each child contributed several datapoints. Model stability was assessed by comparing the estimates obtained from the model based on all data with those obtained from models with the levels of the random effects excluded one at a time (Nieuwenhuis et al., 2012). There were no issues with model stability (Tables S7 and S8). As an overall test of the effect of ATU type we compared each full model with a null model lacking the test predictor but keeping age and the same random effects structure as the full model (Forstmeier & Schielzeth, 2011) using a likelihood ratio test (Dobson, 2002). The data and script for Experiments 1 and 2 can be found on the OSF website: https://osf.io/d3pz5/?view_only=ba368e675f324a56a7cad600d6c39581.

### Results

Table 2 provides an overview of the ATU and Success scores of the tasks. For a detailed breakdown of how children attempted to solve the tasks and at which points in the process children got ‘stuck’, see the Supplementary Material. For each task, two or more children were able to spontaneously, i.e. unaidedly and within a very short time span of 3 min, engage in ATU and solve the task. The double-case ZLS standard requires that for relatively less complex behaviours, which have some (low) probability of occurring by chance, two independent, naive individuals must demonstrate the behaviour. According to this standard, this allows ZLS researchers to be confident enough to conclude that the behaviour was re-innovated from the species’ ZLS, rather than being a chance occurrence (Bandini & Tennie, 2017; see also Supplementary Material for more details). This is the case here. In addition, the materials and problems in our tasks were rather unfamiliar to the participants. Together, these results support the view that simple ATU behaviours as presented here are within the human ZLS and do not rely on social learning to be acquired.

**Table 2.** Number (and percentage) of valid trials in which ATU, correct success and incorrect success were scored in Experiments 1 and 2

	Experiment 1				Experiment 2			
ATU type	Task (*n*_valid trials_)	Associative tool use	Correct success	Incorrect success	Task (*n*_valid trials_)	Associative tool use	Correct success	Incorrect success
Tool set use	Open and probe (33)	23 (69.8%)	18 (54.5%)	2 (6.1%)	Box (40)	6 (15.0%)	4 (10.0%)	4 (10.0%)
	Tube task (30)	8 (26.7%)	3 (10.0%)	1 (3.3%)	Tube (38)	9 (23.7%)	7 (18.4%)	0 (0%)
Metatool use^a^	Sponge push–pull (33)	8 (24.2%)	7 (21.2%)	0 (0%)				
Sequential tool use	Stick stick (32)	2 (6.3%)	2 (6.3%)	6 (18.7%)	Box (38)	11 (28.9%)	11 (28.9%)	1 (2.6%)
	Stick stone (34)	2 (5.9%)	2 (5.9%)	4 (11.8%)	Tube (39)	2 (5.1%)	2 (5.1%)	0 (0%)
Multifunctional tool use					Box (40)	11 (27.5%)	11 (27.5%)	0 (0%)
					Tube (40)	31 (77.5%)	30 (75.0%)	0 (0%)
*N*_total_	162 trials from 66 children	44 (27.2%)	32 (19.7%)	13 (8.0%)	235 trials from 119 children	70 (29.8%)	65 (27.6%)	5 (2.1%)

a Data for Anvil prop not shown here as task was removed from the analysis owing to design failure.

We examined whether the two tasks within the Tool set use type were of comparable difficulty. We found that the Open and Probe task had higher rates of ATU than the Tube task (*χ*^2^(1) = 9.98, *p* = 0.001) as well as higher Correct success rates (*χ*^2^(1) = 12.10, *p* < 0.001), indicating that the Tool set use tasks were not of equal difficulty. We also compared the two tasks within the Sequential tool use type and found them to be of equal difficulty regarding the ATU and Correct success rates (Fisher's exact tests, both *p* = 1.000). As we noticed during data collection that there was a design flaw with the Anvil prop task (the majority of children solved the task in a way not intended by us and not involving ATU), we excluded Anvil prop from all analyses. Therefore, Metatool use was not included in this analysis.

We examined whether ATU type affected children's ATU and Correct success rates. The models comprised 144 trials from 63 children (cases of Incorrect success were removed from the analysis). ATU type had a significant positive effect on ATU rates (*χ*^2^(2) = 30.70, *p* < 0.001). Specifically, Tool set use yielded significantly higher ATU rates than Sequential tool use (*p* < 0.001) and Metatool use (*p* = 0.030; note that Metatool use only consisted of the Sponge push–pull task). The odds for children in the Tool set use condition engaging in ATU over the odds of children in the Sequential tool use condition were 15.01 (95% CI [6.16; 100.38]). The odds for children in the Tool set use condition scoring ATU over the odds of children in the Metatool use condition were 4.05 (95% CI [1.57; 14.79]). Performance in the Sequential tool use and Metatool use tasks was equally low (*p* = .135; Table S7). ATU type also had a significant positive effect on Correct success rates (*χ*^2^(2) = 15.08, *p <* 0.001). Specifically, Tool set use yielded significantly higher Correct success rates than Sequential tool use (*p* = 0.003). The odds for children in the Tool set use condition scoring Correct success over the odds of children in the Sequential tool use condition were 8.98 (95% CI [2.86; 35667.67] – but note the large uncertainty). No other comparisons were statistically significant (Table S8).

### Discussion

Experiment 1 investigated whether 3.5- to 4-year-old children were able to independently re-innovate how to use two tools in different combinations to solve several problem-solving tasks. To our knowledge, this is the first time several ATU types have been investigated in children in a single study. We found that children succeeded in all three tested types of ATU individually, without the need for social learning immediate to the experimental context (note that past social learning could still matter). However, success rates were low, with only one task (Open and probe (Tool set use)) having a success rate of more than 50%, suggesting that the individual invention of these behaviours is – while not impossible – quite challenging for 3- to 4-year-old children. Sequential tool use was especially challenging (~6% Correct success rate). This might be due the fact that the Sequential tool use tasks involved two apparatuses, which might have increased general task difficulty compared with the other ATU types. However, despite this, our tasks were still relatively easy versions of Sequential tool use: we presented only one initial tool (rather than a choice of tools), with which only one other tool could be retrieved. The cost of retrieving the second tool was relatively small and the two apparatuses were in proximity (in contrast, in some studies involving New Caledonian crows, apparatuses are positioned opposite each other (Wimpenny et al., 2009) or completely out of the sight of the others (Gruber et al., 2019) so that subjects have to keep the necessary information in their short-term memory, providing strong evidence of New Caledonian crows mentally representing stages of the problem). The different types of ATU were not equally easy, with Tool set use being the easiest type of ATU, and Sequential tool use possibly the hardest. This could be interpreted as a first hint at a potential ‘cognition-based hierarchical organization’ (Shumaker et al., 2011, p. 21) of ATU. Yet, our results should be treated with caution until future research has been carried out.

Experiment 2 followed up on the question whether Sequential tool use actually posed more cognitive demands than Tool set use tasks or whether the finding from Experiment 1 was caused by task-specific effects. In Experiment 2, we used the *same* apparatus to investigate children's spontaneous engagement in Tool set and Sequential tool use behaviours, removing the confound of task-specific influences on performance. To also investigate potential age effects on performance, we tested a slightly wider age range (3–6 years). Experiment 2 also involved a condition requiring Multifunctional tool use (i.e. using a single tool in different functions) to contrast against Tool set and Sequential tool use.

## Experiment 2

**Figure 3.**
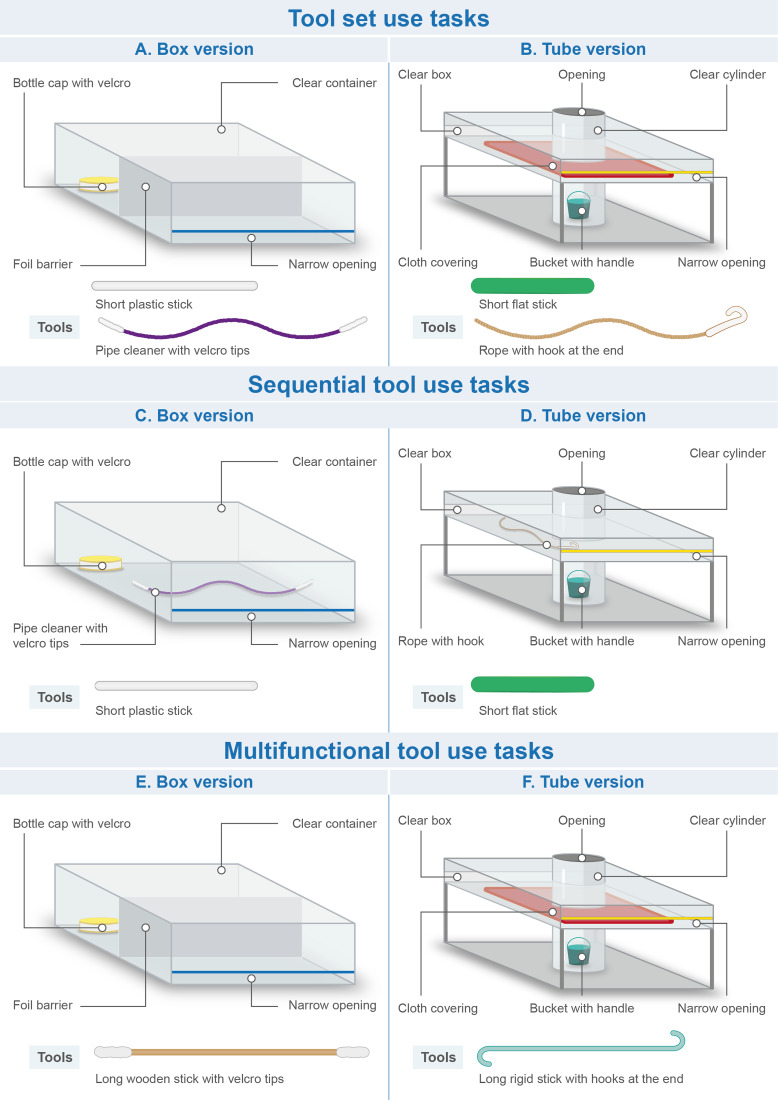
Materials used in Experiment 2. Figure drawn by Nuria Melisa Morales García.

We used two apparatuses (*box apparatus* and *tube apparatus*; Figure 3), which could be presented in two ATU types (Tool set use, Sequential tool use). Presenting the ATU versions on two apparatuses and comparing children's performance across them was a first step towards disentangling effects based on the cognitive demands of the respective ATU type from more task-specific demands (e.g. differences in transparency of the apparatuses, or how easily alternative solutions could be found). A third presentation mode was Multifunctional tool use (described below, but not a major focus of this paper). Children were randomly assigned to one of these three tool-use types (Tool set use, Sequential tool use, Multifunctional tool use). Participants completed two trials within their tool-use type, one with the box and one with the tube apparatus (order counterbalanced).

### Methods

#### Participants

The final sample included 119 children (53 boys) between 3 years 0 months and 6 years 10 months (Mean ± sd: 5 years 0 month ± 12.38 months) tested in nurseries, schools and a Science Museum in Birmingham, UK (*n* = 95) and a nursery in Jena, Germany (*n* = 24) between January and March 2015. The ethnic background of the sample was mainly White (*n* = 109; 91%), nine children (7%) were Black, and one (2%) was Asian. Three additional children were tested but removed from the sample because they were below the age of 3 (*n* = 2) or because of interference from nursery staff (*n* = 1).

#### Material

We used two apparatuses which could be administered in each of the three tool-use versions (Tool set, Sequential, Multifunctional tool use; Figures 3 and S7). For space reasons, tasks are only briefly described here; a full description including animations can be found in the Supplementary Material.

##### Box apparatus – Tool set use

This task was similar to the Open and Probe task of Experiment 1. The goal was to insert a short, sturdy stick into the box to make a hole in a tin foil barrier and to then use a pipecleaner to reach through the hole to retrieve a reward.

##### Box apparatus – Sequential tool use

In this version, there was no barrier inside the box. The tools were the same as in Tool set use (short stick, pipecleaner). The pipecleaner was placed inside the box at the place where the barrier was positioned in Tool set use. The task required using the stick to retrieve the pipecleaner from the box, followed by using the pipecleaner to retrieve the container with the reward.

##### Box apparatus – Multifunctional tool use

As in Tool set use, the interior of the box was divided by a tin foil barrier. However, only one tool was available, combining the functions of the tools in the Tool set use version: a wooden stick covered with hook and loop fastener at both ends, which could both pierce the tin foil barrier and reach and retrieve the container.

##### Tube apparatus – Tool set use

The task was similar to the Push and hook task of Experiment 1. The goal was to insert a stick into the apparatus to remove a cloth barrier by pushing or pulling it via the top or side openings, followed by inserting a rope into the top to hook a bucket with a sticker.

##### Tube apparatus – Sequential tool use

The tools were the same as in Tool set use, but there was no cloth barrier inside the apparatus. The rope was placed inside the box, close to the side facing the participant. The task required using the stick to retrieve the rope via the side openings, after which the rope could be inserted into the top to hook the bucket.

##### Tube apparatus – Multifunctional tool

The setup was as in Tool set use, but there was only one tool available combining the functions of the two tools in Tool set use: a plastic stick with two hooks facing in opposite directions which could both remove the barrier and hook the bucket.

#### Design and procedure

The procedure was similar to Experiment 1, with the difference that here participants were given 4 min to solve the task (for a full description of the procedure, see Supplementary Material).

#### Scoring and analysis

Children's behavior was live-coded. We recorded whether the tool(s) associated with each task were picked up and used in the manner intended by the experimenter. For Sequential tool use and Tool set use we again scored ATU and Correct success. In the Multifunctional tool use task, we scored whether the tool was used in both its intended functions (equivalent to ATU) and Correct success. We also scored instances of Incorrect success for all tasks. To obtain inter-observer reliability, 84% of the valid trials were coded by a second rater (68% of these trials were coded live, the rest from video). Interrater agreement for Correct success was very good (Cohen's *k* = 0.988).

All 119 children participated in two tasks, resulting in 238 trials. Three trials had to be removed from the analysis: in the Tool set use version of the Tube task, one trial had to be excluded as the cloth did not fully cover the hole and another trial had to be excluded because the child cried. In the Sequential tool use version of the Box task, one trial had to be excluded because the child tipped the box. We first carried out descriptive analyses. Then we examined whether the two versions within each tool use type were of comparable difficulty regarding ATU and Correct success by conducting McNemar tests (with continuity correction). To investigate the effects of condition and age on ATU and Correct success, we ran two GLMMs with binomial error structure and logit link function. Age in months (*z*-transformed) and condition were entered as predictor variables, and participant ID was included as a random effect.

### Results

Table 2 provides an overview of the ATU and Success scores of the tasks (Table S9 provides the same overview split by age groups). Out of the 116 participants who completed two valid trials, 60 children (52%) were unsuccessful in both tasks, 44 children (38%) had one of the tasks correct and only 12 children (10%) were able to succeed in both tasks, suggesting that the tasks were challenging for the children. Despite the low success rates, especially in the youngest age group (Table S9), children as young as 3 years of age were able to independently re-innovate Tool set and Sequential tool use. The low success rates could not be explained by a lack of motivation as children were generally found to be interested in the tasks and interact with the tools: In each condition, at least 82% of the children picked a tool up (see Tables S10–S15). A more detailed overview of how children attempted to solve the tasks can be found in the Supplementary Material.

In the Tool set use condition, there was no significant difference between the box and tube versions for either ATU (*p* = 0.579) or Correct success (*p* = 0.505). Both versions were very challenging, partly owing to task-specific reasons that are not necessarily tied to the ATU nature of the tasks: in the box version, while most children correctly inserted the stick into the box, many did not pierce the tin foil barrier – either because they tried to reach the reward by pushing the stick over the barrier or because they failed to pierce the foil despite trying (Figure S6). Note that this difficulty in piercing the tin foil did not occur in the Open and probe task of Experiment 1. This was possibly because (a) it was not possible to bypass the tin foil barrier in the Open and probe task and (b) in Experiment 2 we used a larger piece of tin foil, which might have resulted in the foil being held in the cardboard frame more loosely, making it harder to break it. Similarly, in the tube version, many children accidentally dropped the stick into the tube or failed to remove the cloth barrier despite trying (Figure S7). If these design aspects made the successful use of the first tool of the tool set (i.e. the stick) difficult, it means that Experiment 2 could potentially underreport children's ATU capacities in a Tool set use context.

In the Sequential tool use condition, the box version yielded significantly higher rates of ATU (*p* = 0.027) and Correct success (*p* = 0.016) than the tube version. This was possibly because many children did not notice the rope inside the tube apparatus and/or accidentally dropped the stick into the tube, which posed additional challenges to the tube version (see the Supplementary Material for a longer discussion).

In the Multifunctional tool use condition, the tube version revealed significantly higher rates of ‘ATU’ (i.e. using the tool correctly in both functions) and Correct success than the box version (both *p* < 0.001). In the box version, the challenges were similar to the box version of the Tool set use condition: many children either failed to break the tin foil barrier despite attempting and/or tried to reach the reward by pushing the tool over the barrier. The tube version, however, was easier in the Multifunctional tool use condition as it was not possible to drop and lose the tool in the tube (owing to its length) and because it seemed to be easier to remove the cloth barrier. In sum, the challenges the children faced were not only determined by the tool use condition, but also in large part by task-specific aspects.

The GLMMs consisted of 235 observations from 119 children. Regarding the model with ATU as a dependent variable, age and condition together explained the data significantly better than a null model only consisting of the intercept (*χ*^2^(3) = 47.14, *p <* 0.001). Condition had a significant positive effect on ATU rates (*χ*^2^(2) = 33.98, *p <* 0.001; Table S16). While Tool set and Sequential tool use did not differ in their ATU rates (*p* = 0.998), they were both significantly more difficult than Multifunctional tool use (both *p* < 0.001). The odds for children in the Tool set use condition scoring ATU over the odds of children in the Multifunctional tool use were 0.16 (95% CI [0.06; 0.32]), i.e. the odds of scoring ATU in the Tool set use condition were decreased by 84% compared with the Multifunctional tool use condition. The odds for children in the Sequential tool use condition scoring ATU over the odds of children in the Multifunctional tool use were also 0.16 (95% CI [0.05; 0.34]), i.e. also decreased by 84%. Age had a significant, positive effect on ATU rates (*χ*^2^(1) = 14.68, *p <* 0.001). With each month increase, there was an increase in the odds of scoring ATU of 1.85 (95% CI [1.37; 2.77]).

This pattern of results was similar when using Correct success as dependent variable. Age and condition together explained the data significantly better than a null model only consisting of the intercept (*χ*^2^(3) = 55.51, *p <* 0.001). Condition had a significant positive effect on Correct success rates (*χ*^2^(2) = 37.52, *p <* 0.001; Table S17). As with ATU, the Correct success rates in the Tool set and Sequential tool use conditions did not differ (*p* = 0.635) but were significantly lower than in Multifunctional tool use (both *p* < 0.001). The odds for children in the Tool set use condition scoring Correct success over the odds of children in the Multifunctional tool use were 0.11 (95% CI [0.03; 0.23]), i.e. the odds of scoring Correct success in the Tool set use condition were decreased by 89% compared with the Multifunctional tool use condition. The odds for children in the Sequential tool use condition scoring Correct success over the odds of children in the Multifunctional tool use were 0.17 (95% CI [0.06; 0.33]), i.e. they were decreased by 87%. Age had a significant, positive effect on Correct success rates (*χ*^2^(1) = 21.11, *p <* 0.001). With each month increase, there was an increase in the odds of scoring Correct success of 2.20 (95% CI [1.58; 3.51]).

### Discussion

Experiment 2 investigated children's performance in Tool set and Sequential tool use tasks and compared it with their performance in Multifunctional tool use tasks. In contrast to Experiment 1, in which the tasks of different ATU types differed in both ATU type and in the apparatus and task design, in Experiment 2 the different conditions were more comparable as they were administered using the same kinds of apparatuses (a box and a tube apparatus, each of which could be presented in each of the three tool use versions).

Supporting the findings from Experiment 1, the results showed that children from 3 years of age were able to independently invent how to use two tools in different combinations to solve these tasks, even when given no familiarisation phase, only 4 min per task and no opportunity for social learning. Despite this, the results again showed that spontaneously re-innovating ATU in novel tasks is difficult for preschoolers, at least under testing situations as used here. We found no evidence that Tool set and Sequential tool use differed in difficulty. However, both tasks were significantly more difficult than the Multifunctional tool use tasks, i.e. tasks which had the same setup as the Tool set use versions but differed in the number of tools provided (a single tool that could be used in two modes). Success rates increased with age over the tested range from 3 to 6 years. A more fine-grained analysis of solution approaches showed that performance was not only affected by the specific tool-use demands of a task (i.e. whether Sequential tool use or the use of a Tool set was required), but also to a large part by the idiosyncratic features of each task, despite the use of the same apparatus across ATU versions.

## General discussion

The aim of the current study was to address the gap in the developmental literature on the emergence and spontaneous invention of ATU behaviours in human children. This study investigated children's performance in tasks involving Sequential tool use (using a tool to get a tool), Metatool use (using one tool to improve a second tool) and Tool set use (using two tools to achieve a single outcome) and showed in two experiments that children from 3 years of age can spontaneously invent solutions to all three of these ATU types individually, i.e. without the need for immediate social learning. These findings suggest that ATU behaviours lie within the human ZLS, as they have also been shown to do for all species of great apes and some other non-human primate and bird species (Table 1 and Table S1 in the OSF repository). The presence of spontaneous ATU in humans and non-human great apes suggests that the last common ancestor of humans and apes, living ~13 million years ago (Stewart & Disotell, 1998), was probably also able to engage in ATU without the need for copying know-how.

We minimised the possibility that children could draw on directly relevant cultural knowledge by using novel tasks that children were unlikely to have encountered before. This does not mean that children did not draw on more general knowledge, e.g. about affordances and the physical properties of the materials involved (e.g. ropes, hooks, pipecleaners). Assuming that the novelty of the tasks and the participants’ relatively young age were effective in minimising children's ability to use specific cultural knowledge, the results suggest that all of the tested ATU types potentially lie within the human ZLS, i.e. at least some types of ATU can be invented without social learning.

Our experiments still need to be replicated in other, non-Westernised cultures to allow for a stronger conclusion about the human ZLS. In addition, future studies could attempt to test even younger children. The age range tested in the current study was slightly higher than what was used in a previous study on simple tool use (Reindl et al., 2016) as it was assumed that ATU would demand greater executive function skills (especially working memory), which are still developing in children (Garon et al., 2008; Reindl et al., 2021).

Across both experiments, success rates were low, supporting previous findings that ATU is challenging for preschoolers (Alpert, 1928; Matheson, 1931; Metevier, 2006). Experiment 2 showed that children's performance still increased with age. Our tasks were purposefully created to be challenging. Children were given only a short time to explore and attempt the tasks, which might have decreased the ecological validity of the task and artificially limited children's tool-using skills. In recent years evidence has accumulated that longer testing times will result in higher success rates in children (Breyel & Pauen, 2021; Voigt et al., 2019). Thus, it is possible that when administering our tasks with a longer time window, more children will find solutions. To increase the ecological validity of such studies, future projects should provide longer testing times and more possible solutions, and arguably also administer tasks to pairs of participants instead to individuals only (see Gönül, Hohenberger, Corballis, & Henderson, 2019; Reindl & Wronski, 2022).

How do children's low success rates compare with the success rates reported so far for non-human animals? Based on the data available to us (Table S1 in the OSF repository), we calculated the percentage of successful participants for all previous ATU experiments for which both the number of tested individuals and the number of successful individuals were reported (note that we did not include studies listed in the ‘questionable cases’ sheet, e.g. those in which individuals received training or had considerable prior experience with parts of the task), resulting in 55 entries across species and ATU types with which we could compare our results (Table S1 in the OSF repository, sheet ‘Success rates’). Success rates vary greatly, even within a single species and ATU type. This is not surprising as studies differed in tasks used, testing time, and in whether alternative solutions to the task were available. However, it seems that the highest success rates were produced in Sequential tool use studies – for all four great ape species, capuchin monkeys, rhesus macaques, baboons and rooks the percentage of successful individuals was substantially greater than 50% (but note that the sample sizes were extremely small) – with the exception of New Caledonian crows, whose success rate (14%) is similar to children's average success rate in the current study (12%). However, it should be noted that these numbers could misrepresent animals’ abilities owing to publication bias and selective testing (i.e. in some studies only those individuals who had solved simple tool use problems were able to advance to ATU tasks, e.g. Metevier, 2006). The task which resembles most closely an already existing task is our Stick stick task (Sequential tool use, Experiment 1), as it was based on Taylor et al.'s (2007) task for New Caledonian crows (see also Taylor et al., 2011; Wimpenny et al., 2009). While the success rate in these three bird studies is at 100% and thus in stark contrast to the results reported here for children, one has to note that the crows had prior experience with one or more parts of the tasks. Therefore, before we can draw firm conclusions about the ATU abilities within and especially between species, further studies need to be conducted that allow more direct comparisons. Future research could adapt tasks that have been used with non-human animals to humans (and other species), and tasks used in the current study could be adapted for use with non-human animals.

Evidence for whether there was a cognitive hierarchy of the three examined ATU types was inconclusive. Experiment 1 showed that Tool set use yielded higher ATU and Correct success rates than Sequential tool use, as well as higher ATU rates than Metatool use, suggesting tentative evidence for the existence of such a hierarchy. However, there might be an alternative hypothesis explaining the differences between Tool set and Sequential tool use: the low ATU and Correct success rates in the Sequential tool use rates could be explained by the fact that both tasks consisted of two separate apparatuses, which might have been an additional source of difficulty for the children. Yet there is no evidence in the literature that the use of (spatially separate) apparatuses or platforms poses an additional cognitive demand in tool use tasks for non-human animals or children (Gruber et al., 2019; Jackson, 1942; Martin-Ordas et al., 2012; Miller et al., 2020; Mulcahy et al., 2005; Warden et al., 1940). Experiment 2 controlled for the number of apparatuses (using a single apparatus for both ATU versions) and found that Tool set and Sequential tool use did not differ in either ATU nor Correct success rates. However, performance in Experiment 2 was found to be substantially affected by task-specific features unrelated to ATU type. For example, in the Tool set use task of the box apparatus, some children failed to engage in ATU because they did not use enough force to pierce the barrier or tried to circumvent it – which might have appeared as a potentially successful strategy as the distance between the tool and the reward was reduced and this might have made it difficult for children to recognise this as a wrong attempt. Similarly, in the Tool set use task of the *tube* apparatus (i.e. same ATU type but different apparatus) many children failed as they accidentally dropped the stick into the apparatus and were unable to retrieve it. These inadvertent design features might have artificially decreased children's Tool set use abilities, and thus hindered the detection of a potential cognitive hierarchy. This emphasises the importance of investigating tool use with a multitude of tasks sharing the cognitive demand in question to be able to describe the emergence and development of these skills abstracted from task idiosyncrasies (Völter et al., 2018).

**Figure 4.**
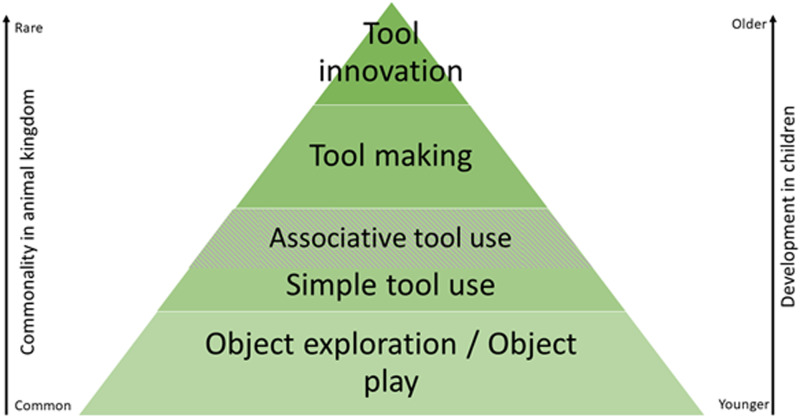
Adapted from Neldner's (2020; Figure 1.1) proposed hierarchy of tool-related behaviours. Here, we explicitly added Associative tool use at the upper end of the Simple tool use category.

While the question whether there is a cognitive hierarchy among the three investigated ATU types requires further research, attempts have been made to create a hierarchy on a broader level, sorting tool use and other tool-related behaviours by their suggested cognitive complexity (Neldner, 2020; Putt et al., 2022; Visalberghi & Fragaszy, 2012). While ATU was not explicitly included in Neldner (2020), we suggest that it could be added at the upper end of the ‘simple tool use’ category to represent an intriguing link to more cognitively challenging tool-related behaviours such as tool innovation (Figure 4). Like simple tool use, ATU ‘requires goal-directed, relational action between multiple objects’ (Neldner, 2020, p. 16), but involves a greater number of objects, posing greater demands on working memory, inhibitory control, causal reasoning and imagination. In contrast to tool innovation, ATU requires no new tools to be imagined; however, it requires imagining the correct sequence of actions and how the objects involved relate to each other. Lastly, ATU is an intriguing addition to such a hierarchy, as it consists of one sub-group of behaviours which links the tool *use* and tool *making* categories, namely *Secondary tool use*.

In Secondary tool use, a tool is used to manufacture (or structurally modify) another tool (Shumaker et al., 2011). It seems to be unique to humans as it has not been reported in any wild non-human animals, and unenculturated, captive chimpanzees have shown no evidence for spontaneous Secondary tool use (Bandini et al., 2021). Although two bonobos and one orangutan were able to make and subsequently use stone tools, this was only after extensive periods of teaching, training and sometimes moulding by humans (Roffman et al., 2012; Schick et al., 1999; Toth et al., 1993; Toth & Schick, 2009; Wright, 1972; for a review see Bandini et al., 2021). Moreover, even after years of practice, the apes did not overcome certain cognitive (and morphological) restrictions to produce tools similar to the earliest hominin stone tools (Toth & Schick, 2009). Secondary tool use is regarded as a major cognitive and technological breakthrough in human evolution which has itself shaped human cognition and culture substantially owing to the coevolution of technological advances and cognitive capacities, such as working memory, planning and technical reasoning (Haidle, 2010; Lombard & Haidle, 2012; Osiurak, 2020; Read, 2008). Future studies could create Secondary tool use latent solution tests for children to find out more about the development of this ATU type.

Tool set and Sequential tool use were more difficult than Multifunctional tool use. This could be because of the larger relational complexity of ATU as, by definition, more objects are involved. This would imply a greater demand on executive function. As inhibitory control, working memory and attention shifting are themselves still developing during childhood and beyond (Best et al., 2009, 2011; Garon et al., 2008), executive function could indeed be a critical developmental bottleneck contributing to the difficulty of ATU for children (for a similar discussion of the importance of working memory for the *evolution* of ATU see e.g. Haidle, 2010; Read, 2017; Read et al., 2021; Wynn & Coolidge, 2014). Indeed, there is evidence suggesting that toddlers’ performance in a tool selection task is correlated with their performance in an inhibition and a short-term memory task (Pauen & Bechtel-Kuehne, 2016) and that preschoolers’ ability to make tools after observing a demonstrator can be predicted by their score on a response inhibition task (S. R. Beck et al., 2016; Gönül et al., 2018). More studies are needed to compare children's performance in ATU and executive function tasks to better understand the cognitive demands involved in ATU and problem-solving in general.

In non-humans, ATU tasks have been used in latent solution tests as well as to study problem-solving and causal cognition more generally. We hope that the tasks introduced here will be a valuable addition to the pool of tests for investigating these and other topics in children and that they will be used and amended in future research.

## Research transparency and reproducibility

The data and script for both experiments presented in this study and Table S1 can be found on the OSF website: https://osf.io/d3pz5/?view_only=ba368e675f324a56a7cad600d6c39581

## Supplementary material

For supplementary material accompanying this paper visit https://doi.org/10.1017/ehs.2022.4. click here to view supplementary material
