# Supplementary material for: Young children spontaneously invent three different types of associative tool use behaviour
Source: Evol Hum Sci. 2022 Feb 2;4:e5. doi: 10.1017/ehs.2022.4 (PMC10426097; doi:10.1017/ehs.2022.4)
Supplement: Supplementary file 1 [file S2513843X22000044sup001.docx]

**Supplementary Material for**

Young children spontaneously invent three different types of associative tool use behaviour

Reindl, E., Tennie, C., Apperly, I. A., Lugosi, Z., & Beck, S. R.

Content:

- Additional methods for Experiment 1
  - Full description of all valid tasks (Animated Figures 1-5, Fig. S1)
  - Description of Anvil prop task
- Additional results for Experiment 1
  - Description of performance in the three conditions, Tables S2-S6; Fig. S2-S6
  - Additional information on the double-case standard by Bandini & Tennie (2017)
  - Tables S7, S8
- Additional methods for Experiment 2
  - Task description (Animated Figures 6-11, Fig. S7)
  - Design and procedure
- Additional results for Experiment 2
  - Descriptive statistics by age groups, Table S9
  - Description of performance in the three conditions, Tables S10-S15, Fig. S8-S13
  - Results from the GLMMs, Tables S16, S17

# **Experiment 1**

## **Full description of all valid tasks**


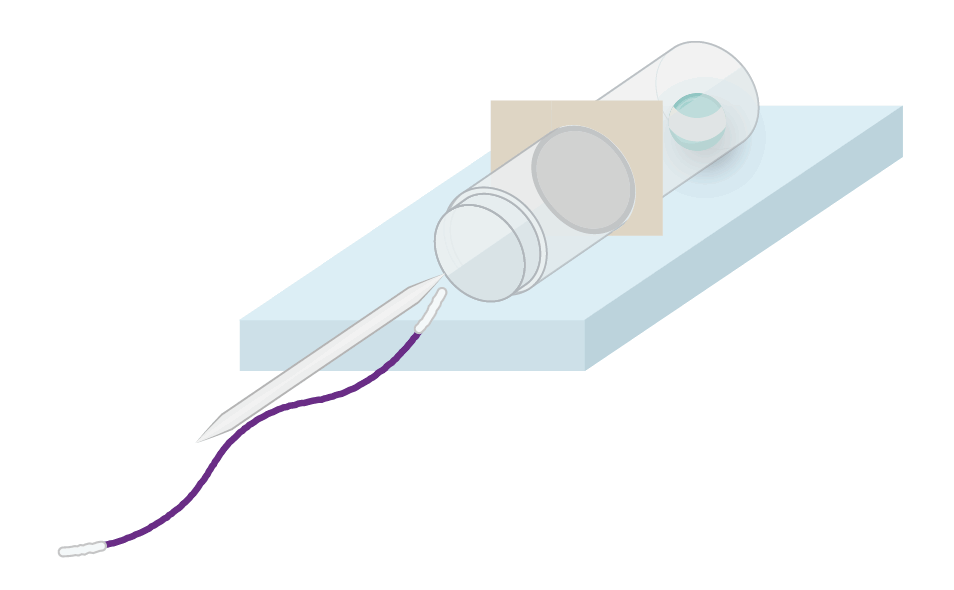
**Tool set use - *Open and probe*.** This task was based on the use of Tool sets by wild chimpanzees to open up beehives, ant nests or termite mounds. It consisted of a transparent horizontal tube (l = 28 cm), at one end of which a ball wrapped in hook and loop fastener was placed (the ball could be exchanged for a sticker). On the other end, there was a round opening (4 cm). In the middle there was a cardboard frame with tin foil serving as a wall blocking access to the ball. Two tools were placed in front of the apparatus: a plastic stick (l = 14.5 cm) made from hand moldable plastic, and a pipecleaner (l = 29 cm) with hook and loop fastener strips attached to both ends. To solve the task, one had to insert the stick into the opening of the tube and pierce the foil in the middle, after which one could use the pipecleaner to reach through the pierced foil into the rear of the bottle to retrieve the ball. The stick could only be used to pierce the foil and was too short to reach the ball. The pipecleaner was too bendy to be able to pierce the foil. The tube was placed in front of the child with the opening facing to the right.

Animated Figure 1. Open and Probe task. Animation created by Nuria Melisa Morales García.

**Tool set use – *Push and hook*.** This task consisted of a transparent T-shaped apparatus (a tube (l = 24 cm, diameter = 8 cm) attached horizontally to a vertical tube (7.5 cm x 7.5 cm x 13 cm)). The goal was to retrieve a bucket containing a sticker from the bottom of the apparatus (similar to the hook task in Chappell et al., 2013). At the intersection of both tubes there was a barrier (a rectangular piece of sponge covered with grey tape, 10 x 6.5 x 1.5 cm) which blocked access to the bottom part of the tube containing the bucket. On both sides of the horizontal tube there were two small openings (b = 7 cm, h = 2.3 cm); the vertical tube had an opening at the top (4 cm). The tools placed in front of the apparatus were a wooden stick (l = 19 cm) and a rope with a hook (l = 41.5 cm). To solve the task, one had to insert the stick into one of the openings and move the block, then one could insert the rope into the top to reach down to the bucket.


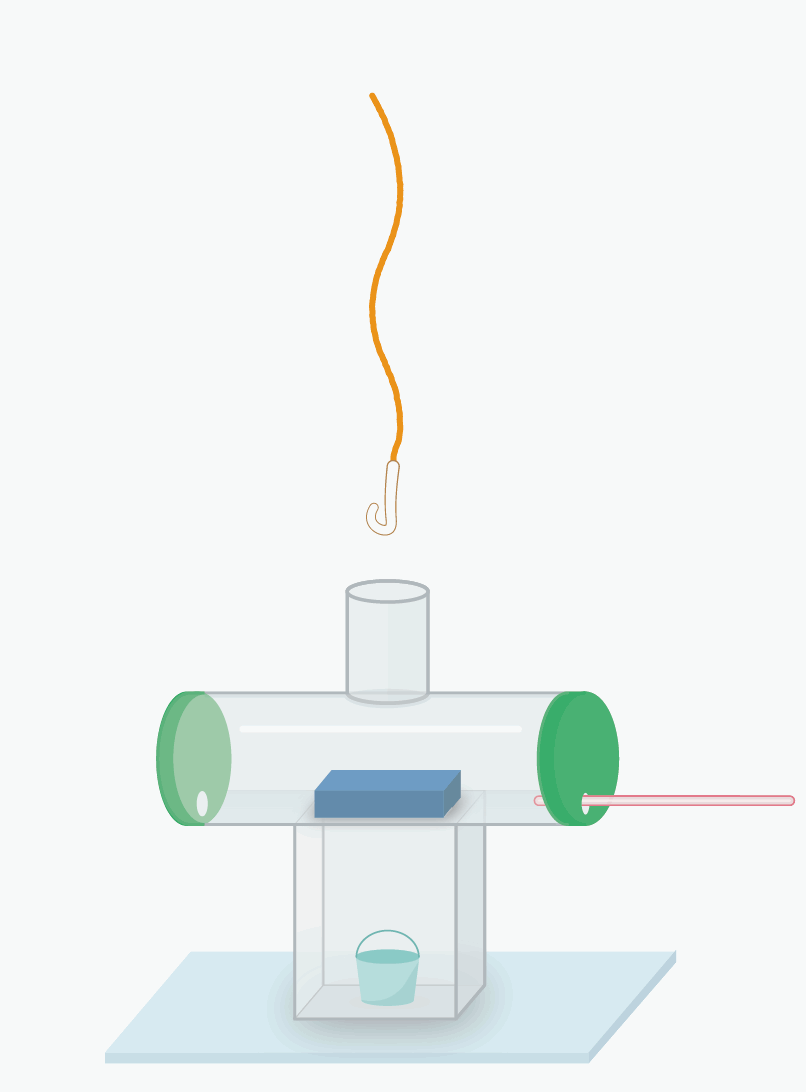


Animated Figure 2. Push and Hook task. Animation created by Nuria Melisa Morales García.

**Metatool use – *Sponge push-pull*.** This task was based on observations of chimpanzees and orangutans using a stick (metatool) to push a leaf/paper towel into a tree-hole or puddle of liquid to more efficiently retrieve water/juice (Matsuzawa, 1991, cited in Sugiyama, 1997; Lehner et al., 2011; Lethmate, 1982). In our task, children were presented with a transparent tube (l = 24 cm, diameter = 7 cm) filled with 500 ml of water and a smaller container placed next to it. The goal was to fill the small container with water. The tools consisted of a wooden stick (l = 47.5 cm, diameter = 0.8 cm) and a ball of cotton wool. While the stick could be used on its own to solve the task – by dipping it into the tube repeatedly to extract water, this method was inefficient. Instead, the stick use could be improved by the wool as a metatool: children could first drop the wool into the tube so that it could absorb water, after which the stick could be used to retrieve the wool. The wool had to be wrung just once to fill the small container. Note that because of the retrieval action (stick used to retrieve wool from bottle), the Sponge push-pull task might resemble a Sequential tool use task, which by definition involves the use of a tool to retrieve another tool. However, the fact that the wool is *out of reach* once it has been used (i.e., dropped into the bottle) and needs to be *retrieved* with the stick is just a feature of this particular task. The retrieval action just happens to be the target action of this task, but is not a feature of Metatool use tasks in general. That is, we could have used a different Metatool use task instead which does not involve a retrieval action as the target action. Sequential tool use is defined as using a tool to acquire another tool. In all existing Sequential tool use tasks this meant that the second tool was out of reach from the start of the experiment (e.g., being far away or enclosed in a container whose inside cannot be reached without a tool, see cases listed in Table S1 [in the OSF repository](https://osf.io/d3pz5/?view_only=ba368e675f324a56a7cad600d6c39581)). However, this is not the case in Sponge push-pull where both the stick and the piece of wool are readily available at the beginning. Furthermore, in Sequential tool use, the second tool is efficient by itself in obtaining the reward – but the first tool is necessary to obtain the second tool in the first place. In Metatool use, the functions of the tools are different. In Sponge push-pull, the stick is not efficient in obtaining the water – but the wool can enhance the stick use (but it is not needed to retrieve the stick).


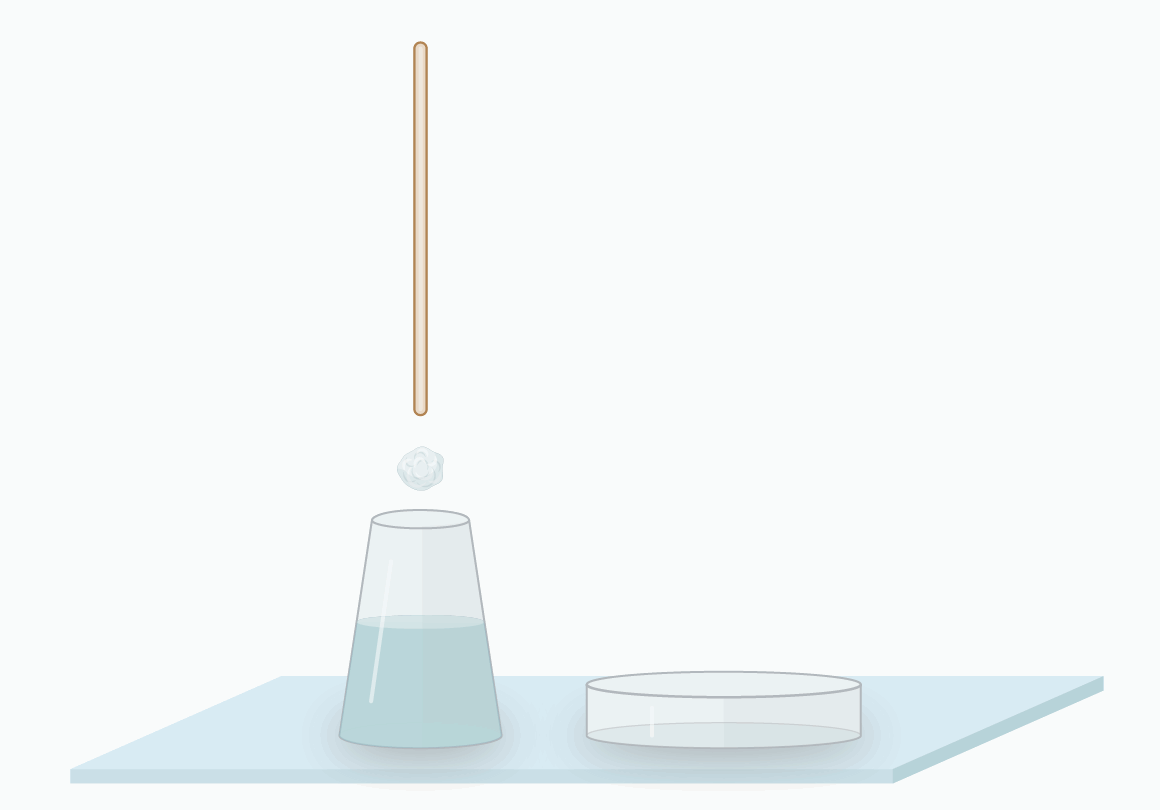


Animated Figure 3. Sponge push-pull task. Animation created by Nuria Melisa Morales García.

**Sequential tool use – *Stick stick*.** This task was based on an apparatus used for studying Sequential tool use in New Caledonian crows (see Fig. 1 in Taylor et al., 2007). In that task, crows were presented with a box containing meat, a bigger box with vertical bars at the front containing a long stick which was needed to retrieve the meat, and a short stick lying in front of the boxes. The birds had to use the short stick to rake the long stick closer to the bars of the big box to be able to retrieve the long stick. Then they could insert the long stick into the small box to extract the meat. Similarly, our task consisted of a small box (30 x 21 x 5 cm) containing a bucket with a sticker and a bigger box with vertical bars at the front and containing a long stick lying at the rear end of the box, opposite of the bars (l = 31 cm). There were no other openings to the big box apart from the front with the vertical bars. A short stick (l = 19.2 cm) was freely available. To retrieve the bucket, children had to use the short stick to rake in the longer stick, then they could use the long stick to obtain the bucket. The small box had a narrow entrance (h = 1 cm) at the front where both tools could be inserted. However, the short stick was too short to reach the bucket, so only the longer stick was functional for retrieving the sticker. The bucket inside the small box was freely movable so that it could be removed from the box with the tool; the box itself was not attached to the table, so in principle children could have picked the box up to make the reward fall out of the apparatus. While this did not happen in our study, in future studies the boxes should be attached to the table in order to prevent children from solving the task in this way.


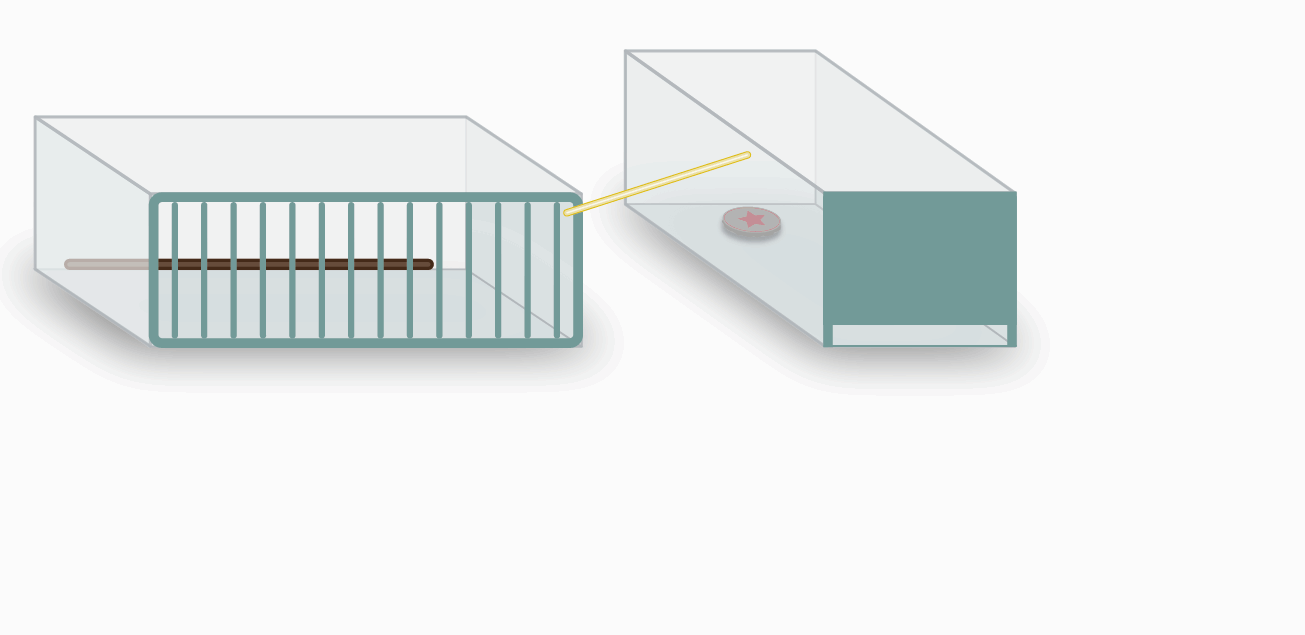


Animated Figure 4. Stick stick task. Animation created by Nuria Melisa Morales García.

**Sequential tool use – *Stick stone*.** This task was the second completely novel task. Metevier (2006) remarked that in most Sequential tool use studies the tools were of the same type (usually sticks of different sizes) and used in a similar fashion (e.g., raking). Therefore, we created a task in which two different kinds of tools (a stick and a stone) had to be used in different ways (pushing, dropping). Children were presented with two apparatuses and a wooden stick (l = 18.5 cm, diameter = 0.4 cm). The task was to insert the stick into the small apparatuses in order to push out a small stone. The stone could then be dropped into the top of the second apparatus where it would activate a trapdoor and release a pom pom. The pom pom could be exchanged for a sticker. The apparatus containing the stone was a transparent L-shaped tube (19 x 6.5 x 7 cm) that was mounted on a base (h = 10.5 cm) in a way that the L was turned 90° clockwise. The horizontal part of the tube had a narrow opening at its side through which the stick could be inserted to push the stone (lying in the middle of the horizontal part of the tube) towards the other end the opening of which was facing the floor. The second apparatus (28 x 20 x 45 cm) was a transparent box with a trapdoor inside held in place by magnets. The pom pom was lying on the trapdoor. Dropping the stone into the apparatus and onto the trapdoor would open the door and release the pom pom. The stick was too short to reach the trapdoor.


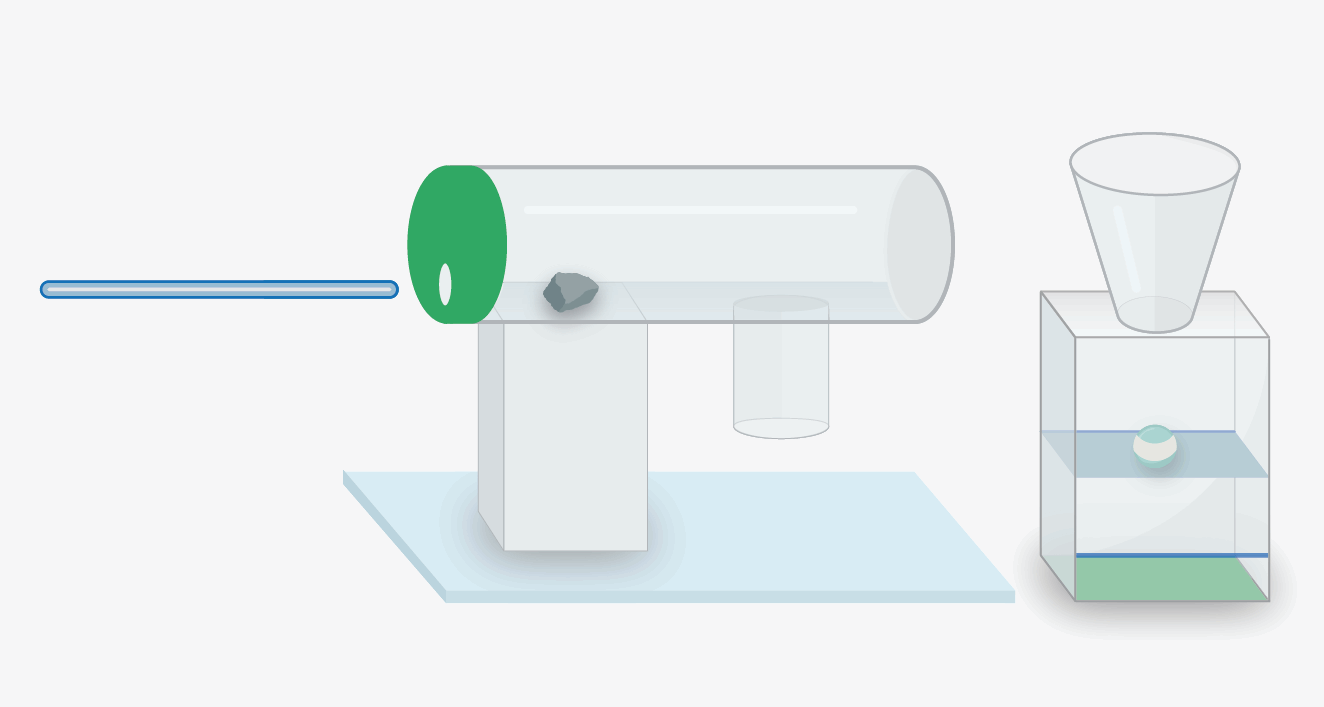


Animated Figure 5. Stick stone task. Animation created by Nuria Melisa Morales García.

| **Tool set use tasks** | |
| --- | --- |
| **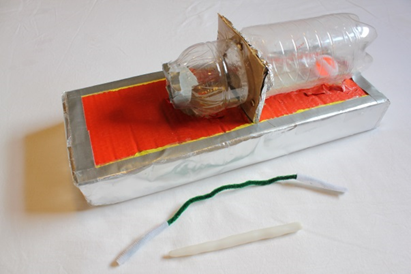**  **Open and probe** | **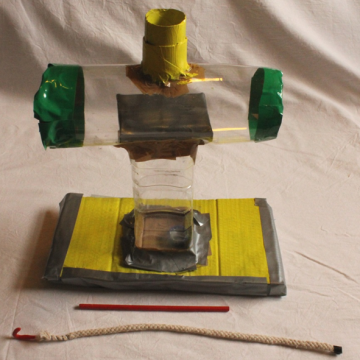**  **Push and hook** |
|  | |
| **Metatool use tasks** | |
| **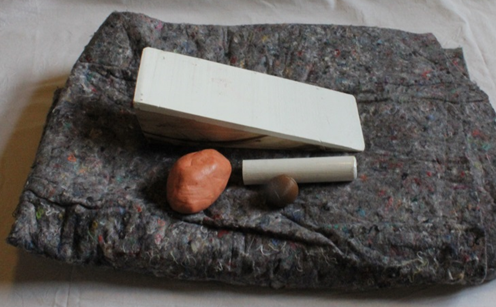**  **Anvil prop** | **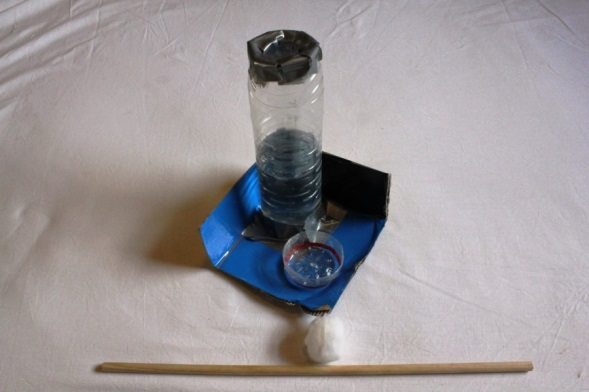**  **Sponge push-pull** |
|  | |
| **Sequential tool use tasks** | |
| **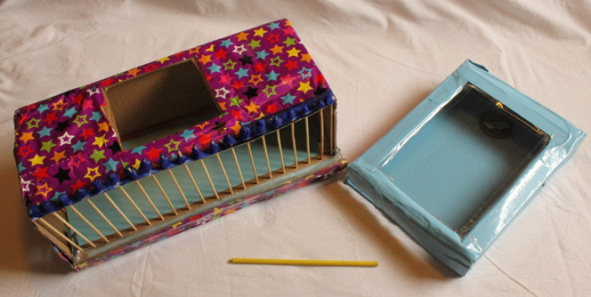**  **Stick stick** | **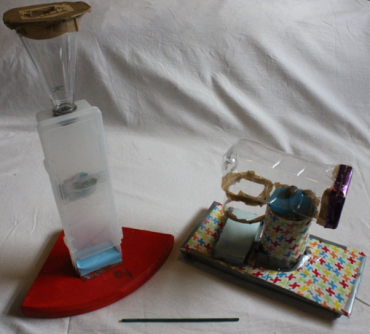 Stick stone** |

Fig. S1. Materials used in Experiment 1. Note: Data from the Anil prop task were excluded from the analysis due to design failure.

## **Description of Anvil prop task**

**Metatool use – *Anvil prop*.** This task was based on the observation of wild chimpanzees engaging in nut-cracking (i.e., using a stone hammer on a stone anvil to crack open nuts) for which sometimes one or even two other stones are used as wedges (metatools) to stabilise the surface of the anvil (Carvalho et al., 2008; Matsuzawa, 1991). In our task, children had to break a plastic “nut” (diameter = 3.5 cm) on a wooden anvil (26 x 10 x 10 cm) using a wooden stick (l = 12.5 cm, diameter = 3 cm) as a hammer. Since the anvil was pyramid-shaped, it was always crooked, regardless of how it was positioned, and thus required stabilisation with a wedge (metatool; a piece of clay, ~ 10 x 6 x 6 cm). A blanket was placed in front of the children, preventing them from using the floor/table as a surface on which they could pound the nut. The anvil was placed on top of the blanket; the rest of the materials were placed between the anvil and the child.

During and after data collection, we judged the task design to have failed: No child scored ATU nor Correct success, but 71% of children were able to open the plastic nut in a way not intended by the task design (Incorrect success): 11 children held the nut in one hand and directly hit it on the anvil; 9 participants positioned the nut on the anvil and held it with one hand while using either the stick (*n* = 7) or the clay ball (*n* = 2) as a hammer. Since alternative ways of solving the Anvil prop task turned out to be readily available to the children, we judged the lack of ATU in this task to not represent a true lack of children’s ATU capacities. Thus, this task was unable to answer the question whether children would be able to solve a Metatool use task, and so we excluded it from further analyses.

## **Description of performance in the three conditions (Tool set use, Sequential tool use, Metatool use)**

### **Tool set use**

In the Open and Probe task, performance was very good. Most children picked up and inserted the stick to pierce the foil and then inserted the pipecleaner in an attempt to retrieve the reward (Table S2). However, only half of the children solved the task unaidedly and in the manner intended by us (Correct success). While children failed at different points in the procedure, a main hurdle seemed to have been a motoric one, namely inserting and maneuvering the pipecleaner through the tin foil barrier after having pierced it (Fig. S1).

The Tube task proved to be more difficult (Table S3). Fewer children picked up the stick; instead, several children selected and used the rope first and never picked up the stick. Therefore, the rope might have stuck out to some children as the obvious correct tool, which might have made it difficult to inhibit using it and switch to using the stick to try to solve the task. There were also two major motoric hurdles: one when inserting the stick to remove the barrier – several children failed to remove the barrier or accidentally dropped the stick into the apparatus; and a second one when trying to hook the bucket with the rope tool (Fig. S2).

Table S2. Descriptive statistics for the Open and probe (Tool set use) task (*n* = 33).

| Picked up stick (at any time) | Picked up stick as first tool | Perforated foil | Picked up pipecleaner (at any time) | Used pipecleaner to reach ball (ATU) | Correct success | Incorrect success |
| --- | --- | --- | --- | --- | --- | --- |
| 29 (88%) | 20 (61%) | 26 (79%) | 27 (82%) | 23 (70%) | 18 (54%) | 2 (6%) |

Table S3. Descriptive statistics for the Push and hook task (Tool set use) task (*n* = 30).

| Picked up stick (at any time) | Picked up stick as first tool | Removed barrier | Picked up rope (at any time) | Used rope to reach bucket (ATU) | Correct success | Incorrect success |
| --- | --- | --- | --- | --- | --- | --- |
| 22 (73%) | 7 (23%) | 12 (40%) | 30 (100%) | 8 (27%) | 3 (10%) | 1 (3%) |


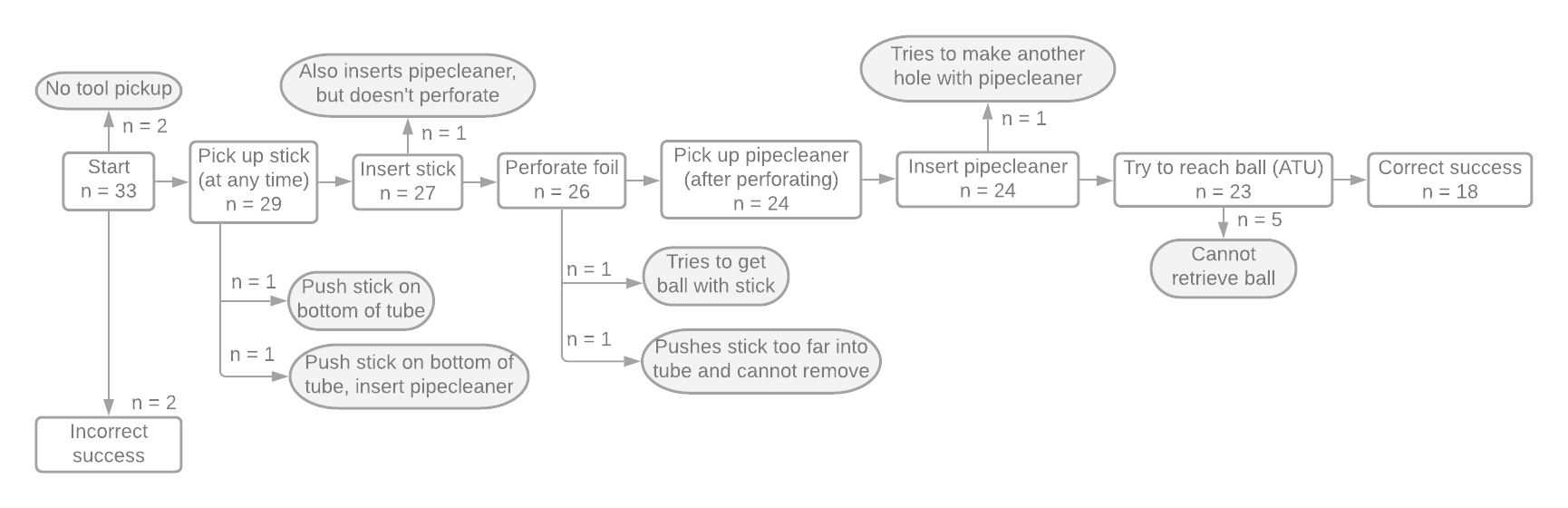


Fig. S2. Number of children at each milestone in the Open and Probe task, with grey fields depicting unsuccessful children. Incorrect success: Both children broke the tin foil with their fingers and then used the pipecleaner to remove the ball.


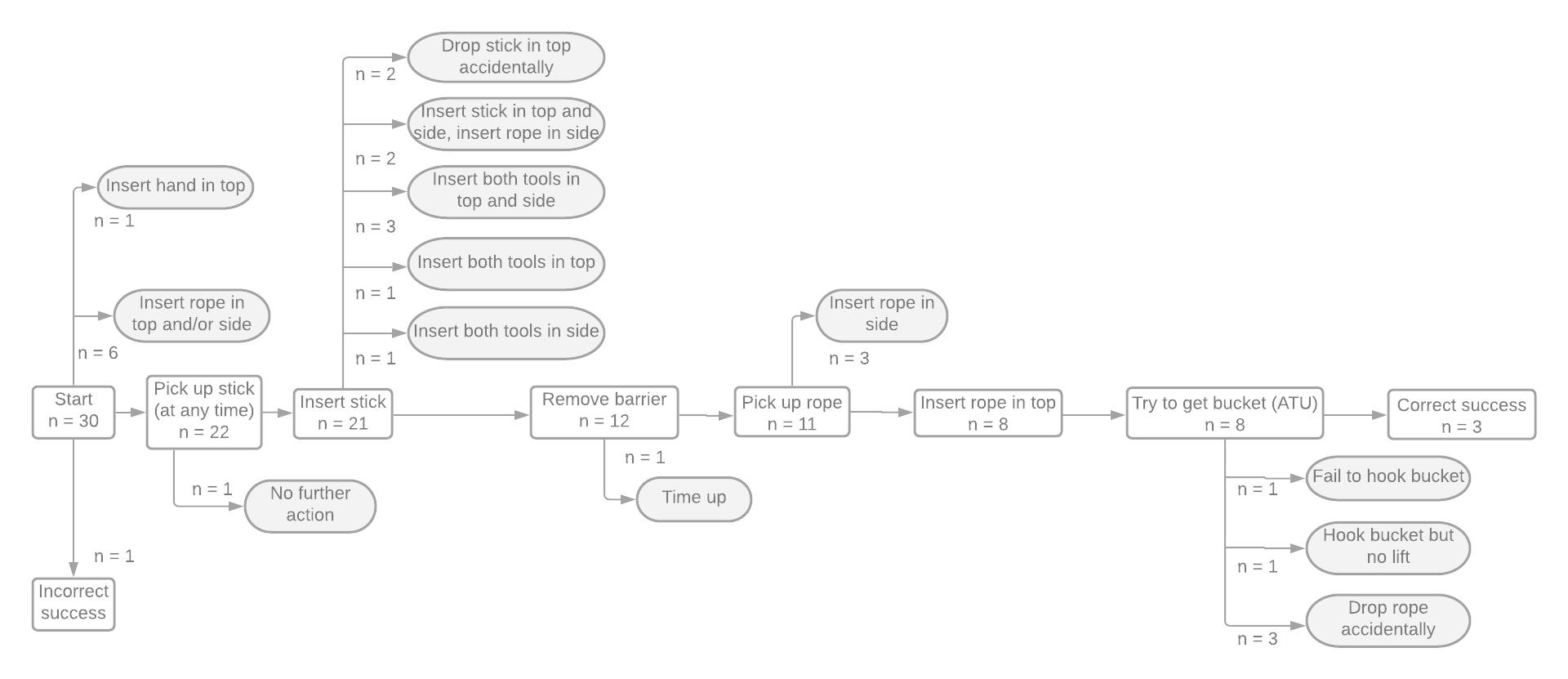


Fig. S3. Number of children at each milestone in the Tube task, with grey fields depicting unsuccessful children. Incorrect success: Child removed the barrier with their fingers and then used the rope to remove the bucket.

### **Metatool use**

In the Sponge push-pull task, both tools were picked up by a majority of the children (Table S4). While most children also inserted the piece of wool into the top of the bottle, only about half of them dropped the wool into the water. Therefore, the major hurdle in this task for children seemed to be to recognize this step as potentially useful in reaching the goal or – if recognized – to decide to drop the wool to let it become out of reach (Fig. S3). All of the children who dropped the wool also inserted the stick afterwards. While some children did not attempt to retrieve the wool, a majority did and was then also successful.

Table S4. Descriptive statistics for the Sponge push-pull (Metatool use) task (*n* = 33).

| Picked up wool (at any time) | Picked up stick (at any time) | Picked up wool as first tool | Dropped wool into bottle | Used stick to reach wool in bottle (ATU) | Correct success | Incorrect success |
| --- | --- | --- | --- | --- | --- | --- |
| 25 (76%) | 29 (88%) | 10 (30%) | 11 (33%) | 8 (24%) | 7 (21%) | 0 (0%) |


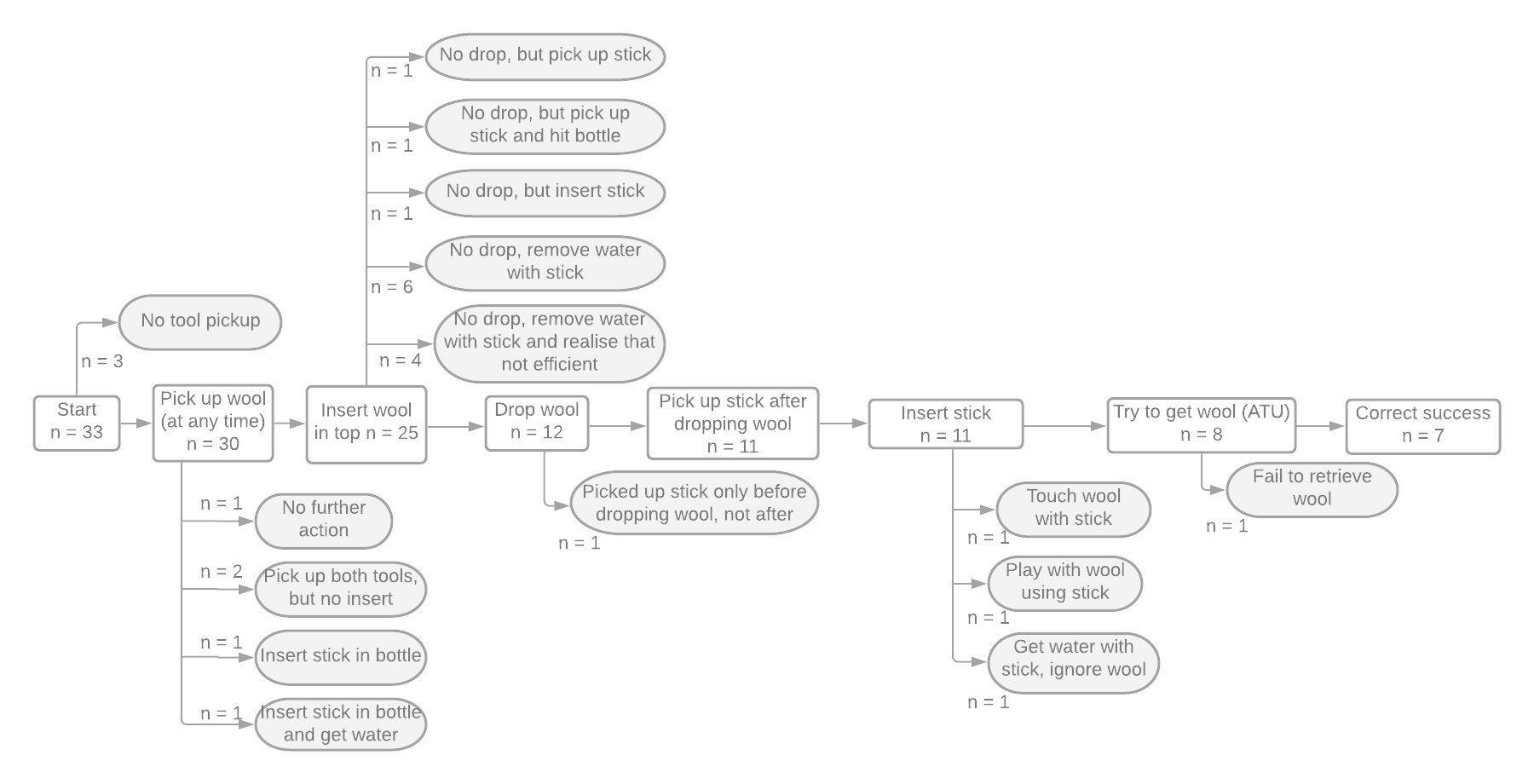


Fig. S4. Number of children at each milestone in the Sponge Push-pull task, with grey fields depicting unsuccessful children.

### **Sequential tool use**

Both Sequential tool use task proved to be very challenging for children (Tables S5, S6). In the Stick-stick task, we had to exclude several children due to poor task design that allowed for Incorrect success. Regarding the remaining sample, while most children picked up the freely available stick, only a small fraction tried to use it to retrieve the longer stick (Fig. S4). Instead, most children tried to reach the reward with the short stick. While some children then accidentally lost the short stick in the apparatus and were unable to obtain it and proceed with the task, a number of children tried reaching the reward with the short stick for the remaining testing time. For those children, the difficulty of the task seemed to be recognising that their attempt might not lead to success or disengaging and trying another strategy.

Similarly, in the Stick-stone task, a large number of children was fixated on retrieving the reward with the stick or their hands and did not use the stick on the second apparatus containing the stone (Fig. S5). Of the five children who obtained the stone, two subsequently ran out of time and could not finish the task, highlighting that a second hurdle for this task was the limited time window.

Table S5. Descriptive statistics for the Stick-stick (Sequential tool use) task (*n* = 32).

| Picked up short stick | Used short stick to get long stick | Obtained long stick with short stick | Used long stick to bucket in box (ATU) | Correct success | Incorrect success |
| --- | --- | --- | --- | --- | --- |
| 29 (91%) | 3 (9%) | 2 (6%) | 2 (6%) | 2 (6%) | 6 (19%) |

Table S6. Descriptive statistics for the Stick-stone (Sequential tool use) task (*n* = 34).

| Picked up stick | Used stick to get stone | Obtained stone with stick | Used stone to get reward (ATU) | Correct success | Incorrect success |
| --- | --- | --- | --- | --- | --- |
| 27 (79%) | 7 (20%) | 4 (12%) | 2 (6%) | 2 (6%) | 4 (12%) |


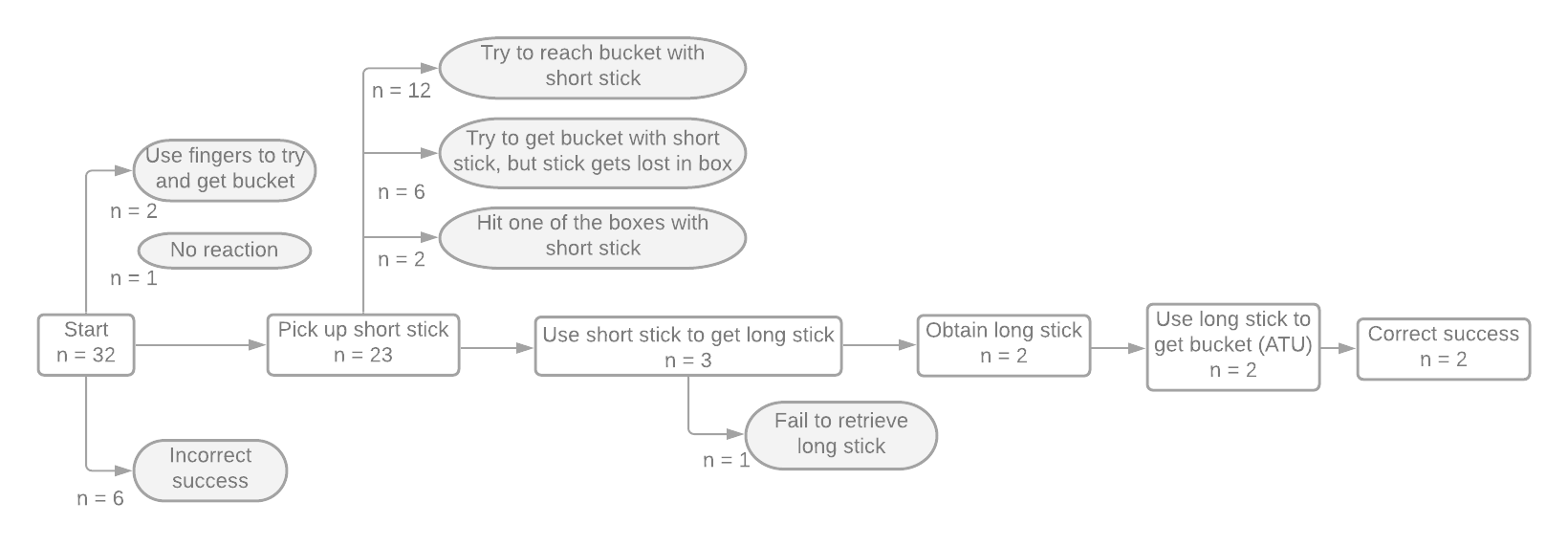


Fig. S5. Number of children at each milestone in the Stick-stick task, with grey fields depicting unsuccessful children. Incorrect success: Children retrieved bucket with hand (4x) or small stick (2x)


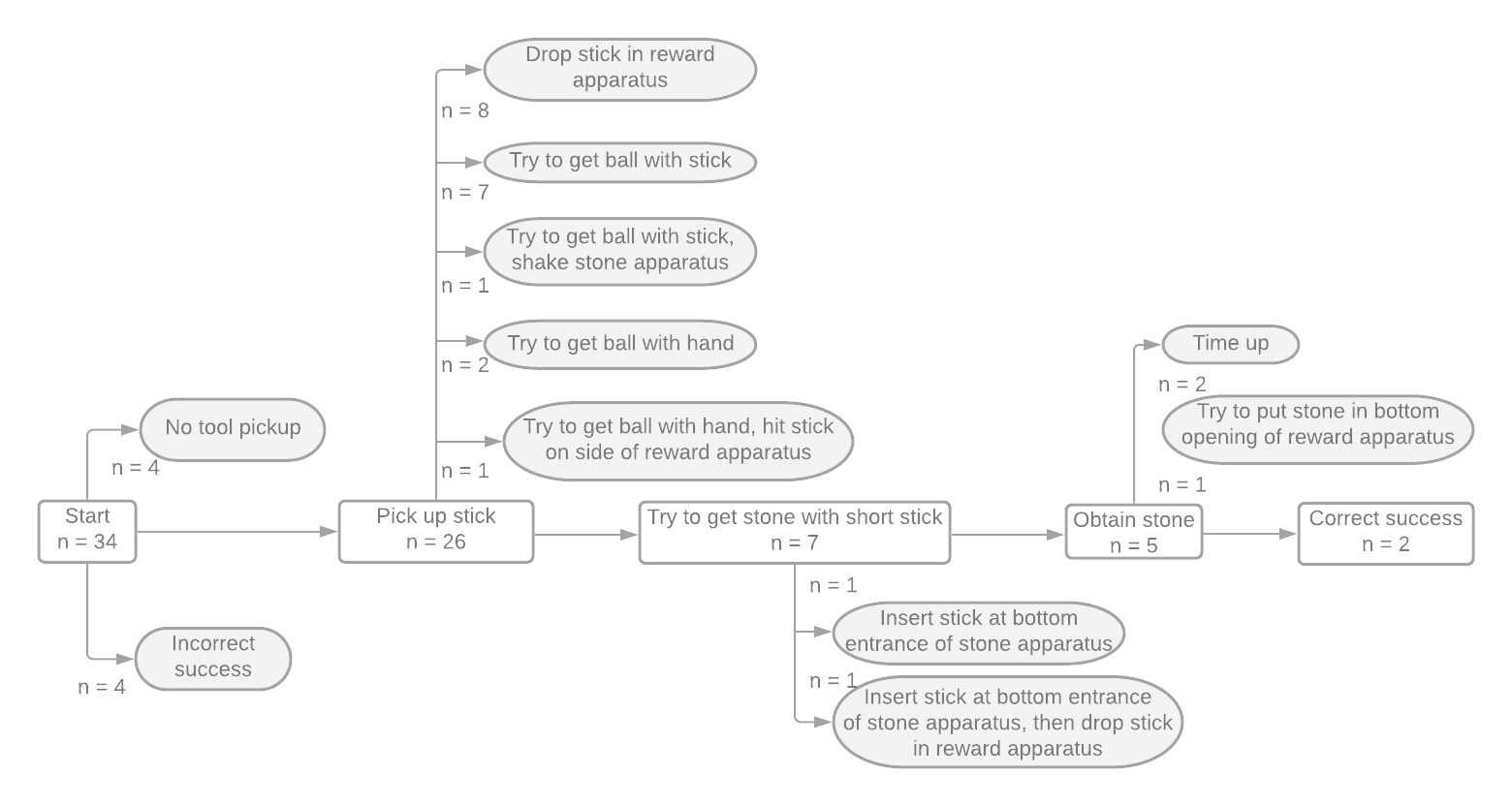


Fig. S6. Number of children at each milestone in the Stick-stone task, with grey fields depicting unsuccessful children. Incorrect success: Children retrieved bucket by dropping stick in apparatus (3x) or by inserting hand in top part of apparatus and pushing the stick onto the trapdoor (1x).

## **Results**

**Additional information on the double-case standard by Bandini & Tennie (2017)**

As a guide for generalising findings from latent solution tests to the species level, Bandini & Tennie (2017) have introduced the *single-* *and double-case standards*. These standards relate to the assumed complexity of the behaviours under investigation. If a behaviour is produced spontaneously by one or several individuals in a latent solution test, it can never be ruled out with full certainty that the behaviour occurred due to pure chance. However, under certain circumstances this probability becomes extremely low. First, the more complex the behaviour of interest is, the less likely is it to emerge purely by chance. This is because more complex behaviours do not consist of just a single action, but of several sub-actions (e.g., picking up a tool, holding and using it in the correct way, bringing it into contact with the target object). In the case of ATU behaviours, an additional difficulty comes from the hierarchical structure of the behaviours (i.e., the target action can be broken down in several sub-goals), requiring actions to be carried out in the correct temporal sequence in order to produce the target behaviour. Each of the sub-actions has a probability to be shown by chance. To estimate chance occurrence for the entire behaviour, the probabilities for the sub-actions need to be multiplied, resulting in smaller probabilities. Of course, the probability that this behaviour is produced in full by mere chance is not zero – this can never be ruled out. Therefore, as a second measure, one can further mitigate the risk of wrongly assuming that an observed is a latent solution rather than a fluke by using the double-case standard. The double-case standard suggests that for simple behaviours at least two independent individuals must spontaneously produce the target behaviour in order for researchers to be confident that the behaviour lies within the species’ Zone of Latent Solutions. For more complex behaviours, a single-case standard can be applied.

The tasks in the current study are all relatively complex, consisting of several sub-steps that need to be carried out in the correct order, and might have qualified for the single-case standard. Yet, we aimed to be conservative and applied the double-case standard. While we can never rule out that behaviours shown by two independent, naive individuals in latent solution tests could have emerged by chance, the complexity of the ATU behaviour, the fact that the tasks were relatively novel to the children, and the use of the double-case standard makes this probability extremely low.

Table S7. Results of the model with ATU as dependent variable (estimates, together with standard errors, bootstrapped confidence limits, significance tests and minimum and maximum of model estimates when excluding individuals one at a time, odds ratios).

| **Term** | **Estimate** | **SE** | **Lower Cl** | **Upper Cl** | **p** | **min** | **max** | **SD** | **Odds ratio [95% CI]** |
| --- | --- | --- | --- | --- | --- | --- | --- | --- | --- |
| Intercept^1^ | -1.297 | 0.482 | -2.486 | -0.521 | ^2^ | -1.537 | -1.225 |  |  |
| Age (in months, z-transformed) | 0.204 | 0.216 | -0.225 | 0.695 | .346 | 0.150 | 0.268 |  | ^4^ |
| ATU type: Sequential tool use vs Metatool use^3^ | -1.310 | 0.687 | -3.238 | -0.047 | = .135 | -1.648 | -1.127 |  | ^4^ |
| ATU type: Tool set vs Metatool use^3^ | 1.399 | 0.552 | 0.454 | 2.694 | **= .030** | 1.307 | 1.676 |  | 4.05 [1.57; 14.79] |
| ATU type: Tool set vs Sequential tool use^3^ | 2.709 | 0.641 | 1.819 | 4.609 | **< .001** | 2.626 | 3.137 |  | 15.01 [6.16; 100.38] |
| Random intercept for ID |  |  |  |  |  |  |  | 0.418 |  |

Notes.^1^The numbers for the intercept are those retrieved with Metatool use as the reference category. ^2^ Not shown due to limited interpretability. ^3^The overall effect of ATU type was χ^2^ = 30.70, df = 2, *p* < .001. ^4^Not shown as not significant.

Table S8. Results of the model with Correct success as dependent variable (estimates, together with standard errors, bootstrapped confidence limits, significance tests and minimum and maximum of model estimates when excluding individuals one at a time, odds ratios).

| **Term** | **Estimate** | **SE** | **Lower Cl** | **Upper Cl** | **p** | **min** | **max** | **SD** | **Odds ratio [95% CI]** |
| --- | --- | --- | --- | --- | --- | --- | --- | --- | --- |
| Intercept^1^ | -1.782 | 0.620 | -15.298 | -0.803 | ^2^ | -2.145 | -1.694 |  |  |
| Age (in months, z-transformed) | 0.309 | 0.283 | -0.224 | 1.010 | .275 | 0.228 | 0.391 |  | ^4^ |
| ATU type: Sequential tool use vs Metatool use^3^ | -1.208 | 0.761 | -9.021 | 0.503 | = .249 | -1.608 | -0.967 |  | ^4^ |
| ATU type: Tool set vs Metatool use^3^ | 0.987 | 0.617 | -0.224 | 7.226 | = .244 | 0.845 | 1.316 |  | ^4^ |
| ATU type: Tool set vs Sequential tool use^3^ | 2.195 | 0.680 | 1.050 | 10.482 | **= .003** | 2.077 | 2.683 |  | 8.98 [2.86; 356667.67] |
| Random intercept for ID |  |  |  |  |  |  |  | 1.102 |  |

Notes.^1^The numbers for the intercept are those retrieved with Metatool use as the reference category. ^2^ Not shown due to limited interpretability. ^3^The overall effect of ATU type was χ^2^ = 15.08, df = 2, *p* < .001. ^4^Not shown as not significant.

# **Experiment 2**

## **Task description**

Box apparatus

The box apparatus consisted of a rectangular cardboard box mounted on four cardboard legs (40 x 30 x 18 cm). The top was transparent to allow visual access to the contents of the box. Along the front was a narrow, horizontal slot (h = 1.5 cm) serving as the only opening of the box. A small plastic container wrapped in hook and loop fastener and containing a sticker was put inside the box, centered at the wall opposite of the opening.

**Tool set use.** This task was similar to the Open and Probe task of Experiment 1. The interior of the box was divided by a barrier of tin foil in a cardboard frame. Two tools were available: A short wooden stick (l = 19.2 cm, diameter = 0.5 cm) and a long white pipecleaner (30cm) whose ends were wrapped with hook and loop fastener. The task required using the stick first to make a hole in the tin foil, followed by using the pipecleaner to reach through the hole to retrieve the container.


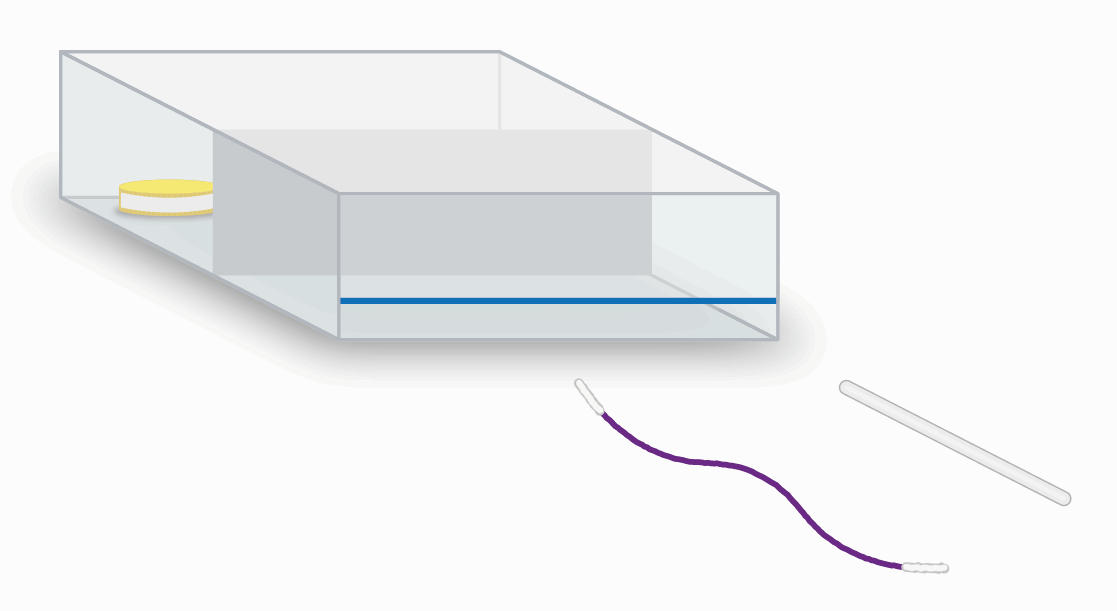


Animated Figure 6. Tool set use – box version. Animation created by Nuria Melisa Morales García.


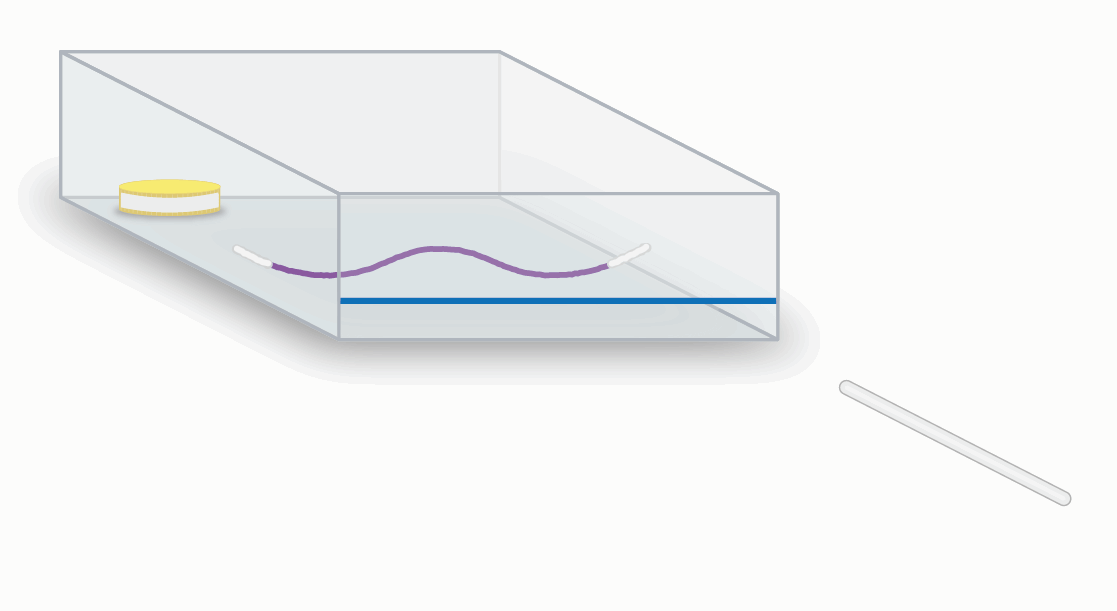
**Sequential tool use.** In this version, the box was not divided by a barrier. The tools were the same as in Tool set use (short wooden stick, long pipecleaner). The pipecleaner was placed inside the box at the place where the barrier was positioned in Tool set use. The stick was placed in front of the box. The task required using the stick to retrieve the pipecleaner from the box, followed by using the pipecleaner to retrieve the container.

Animated Figure 7. Sequential Tool use – box version. Animation created by Nuria Melisa Morales García.

**Multifunctional tool use.** As in Tool set use, the interior of the box was divided by a tin foil barrier. However, only one tool was available, combining the functions of the tools in the Tool set use version: A wooden stick (l = 28 cm, diameter = 0.6 cm) covered with hook and loop fastener at both ends, which could both pierce the tin foil barrier and reach and retrieve the container.


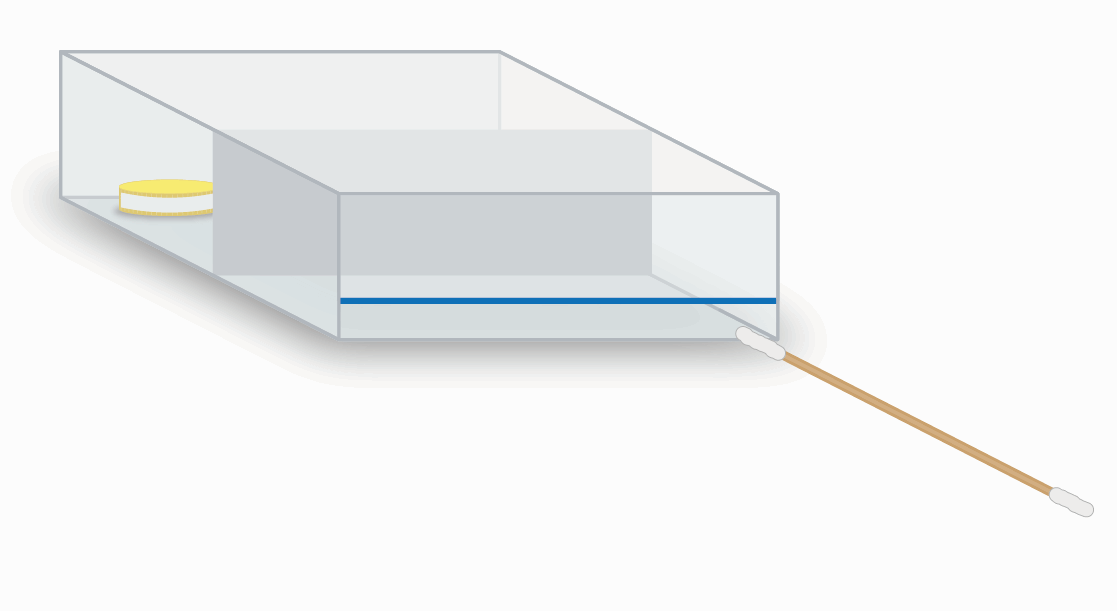


Animated Figure 8. Multifunctional tool use – box version. Animation created by Nuria Melisa Morales García.

Tube apparatus

The tube apparatus was cross-shaped and similar to the Tube task in Experiment 1: It consisted of a transparent tube (h = 17 cm, diameter = 10 cm) onto which a rectangular transparent plastic box (45.5 x 18.5 x 4.5 cm) was mounted horizontally. Attached to the top of the box was a round taper-like opening which allowed access to the content of the tube. The horizontal box had small openings (h = 0.7 cm) on both sides. A bucket with a handle (h = 6.5 cm, diameter = 3 cm) containing a sticker was placed at the bottom of the tube.

**Tool set use.** The opening at the intersection of box and tube was covered by a red piece of cloth (27.5 x 14 cm) used as a barrier. Two tools were available: A short, flat wooden stick (15 x 1.8 x 0.1 cm) and a rope with a hook at one end (l = 32 cm). The task required using the stick first to remove the cloth by pushing or pulling it via the top or side openings, followed by inserting the rope into the top to hook the bucket.


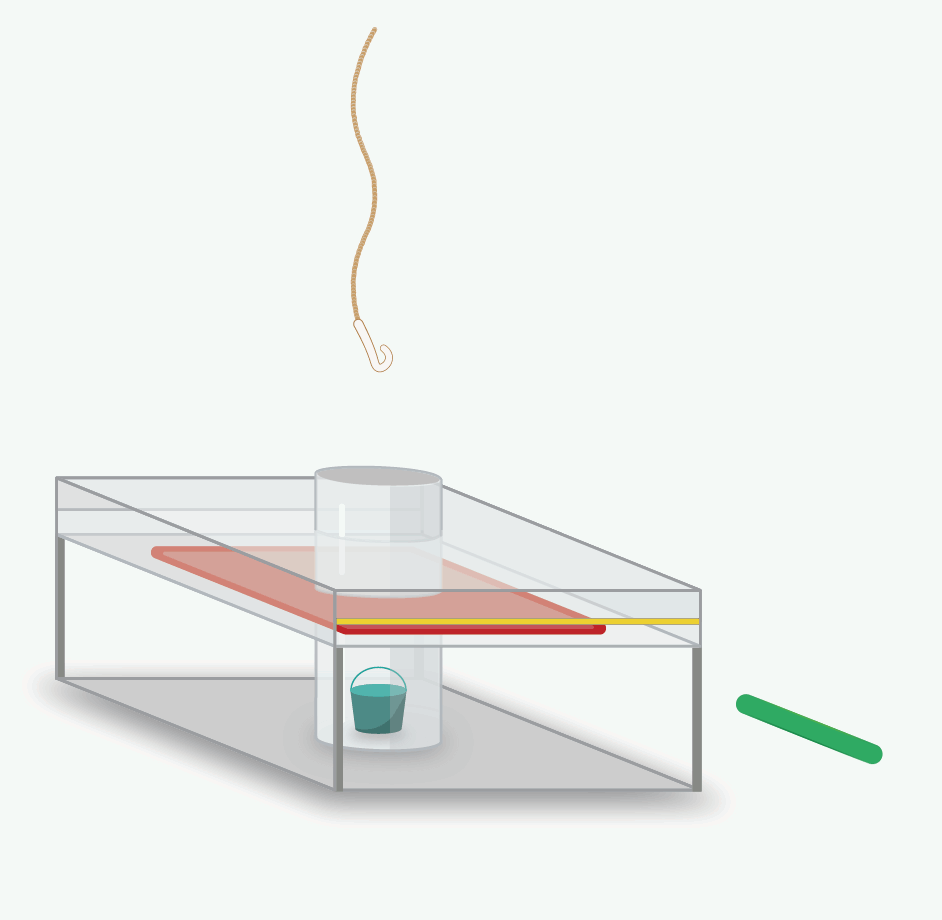


Animated Figure 9. Tool Set use – tube version. Animation created by Nuria Melisa Morales García.


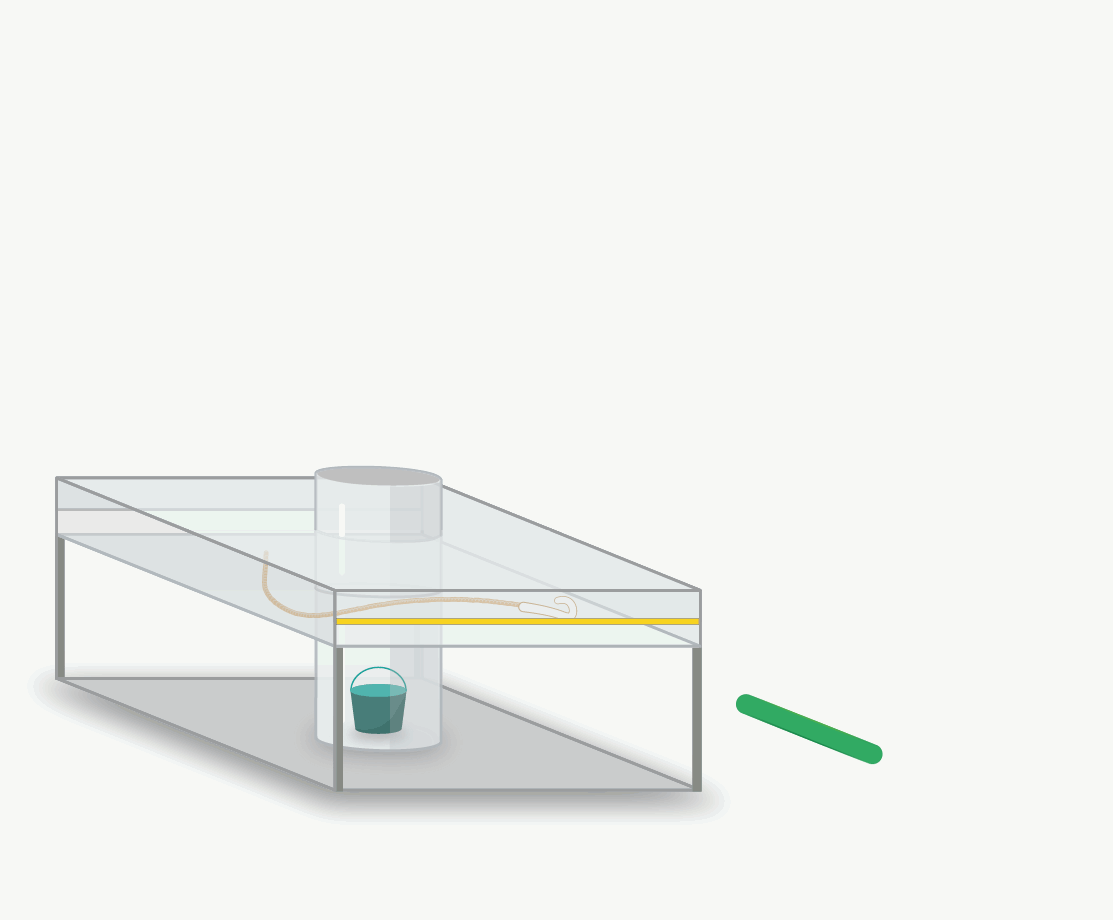
**Sequential tool use.** The tools were the same as in Tool set use, but there was no barrier inside the apparatus. The rope was placed inside the box, close to the side facing the participant. The stick was placed in front of the apparatus. The task required using the stick to retrieve the rope via the side openings, after which the rope could be inserted into the top to hook the bucket.

Animated Figure 10. Sequential tool use – tube version. Animation created by Nuria Melisa Morales García.


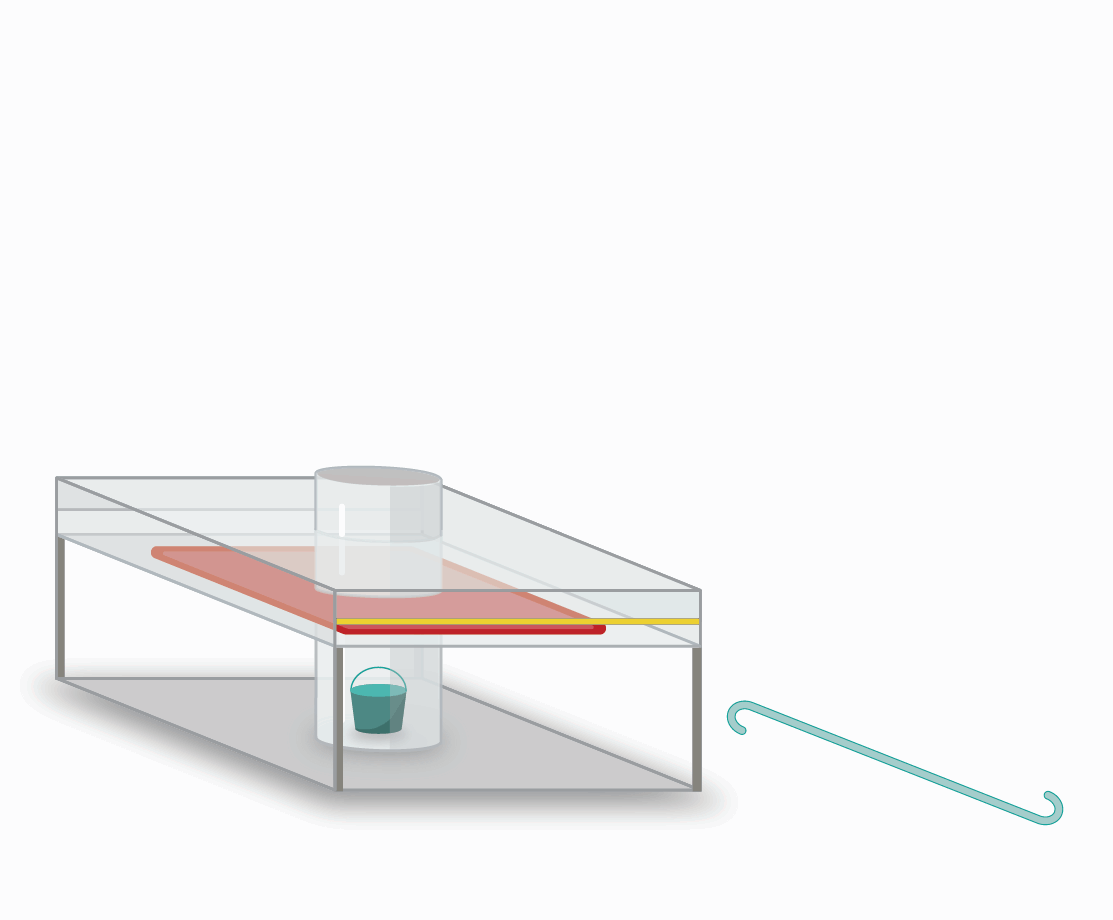
**Multifunctional tool.** As in Tool set use, the intersection of tube and box was covered by the cloth-barrier. Only one tool was available combining the functions of the two tools in Tool set use: A plastic stick (31 x 3 x 0.4 cm) with two hooks facing in opposite directions which could both remove the barrier and hook the bucket.

Animated Figure 11. Multifunctional tool use – tube version. Animation created by Nuria Melisa Morales García.

|  | **Box apparatus** |  | **Tube apparatus** |
| --- | --- | --- | --- |
| **Tool set use** | **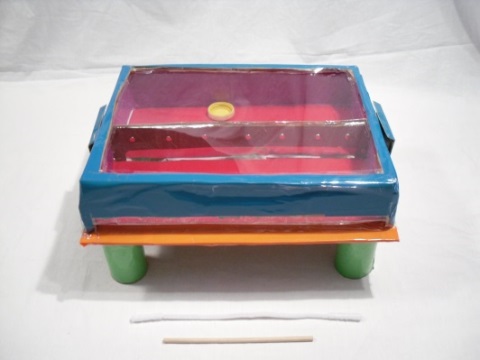** |  | **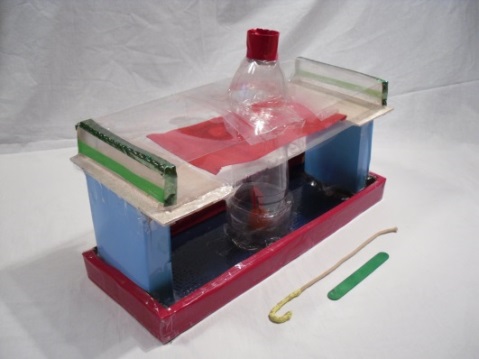** |
| **Sequential tool use** | **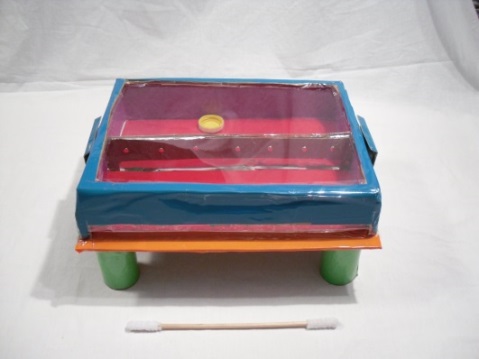** |  | **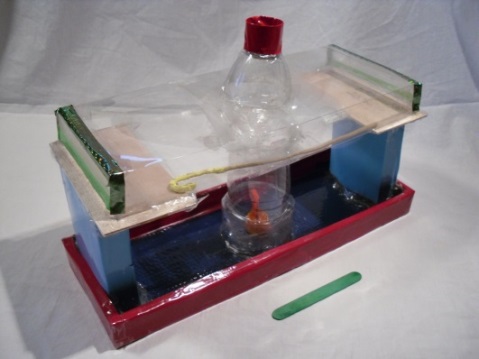** |
| **Multi-functional tool use** | **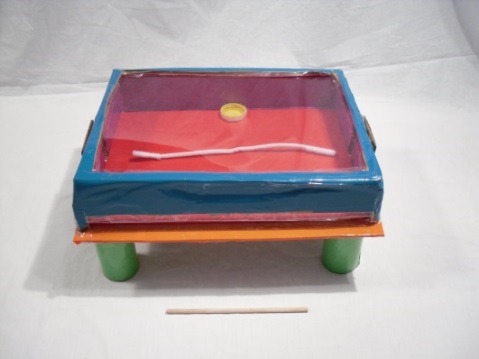** |  | **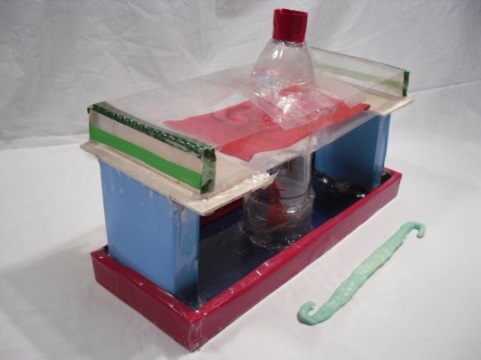** |

Fig. S7. Materials used in Experiment 2.

## **Design and procedure**

The same warm-up game as in Experiment 1 was used. For each task, the experimenter (E.R.) placed the apparatus and the tool(s) in front of the child, with all readily accessible tool(s) lying centrally in front of the apparatus. The experimenter drew the child’s attention to the bucket inside the apparatus: “Can you see the orange/yellow bucket in there? There is a sticker in it!” and explained the goal of the game: “If you can get the bucket out of here, you can keep the sticker! You can use anything here on the floor to help you get the sticker.”. Children were not instructed to use the tools and were only given general encouragement (e.g., “Keep trying!”, “You can do it!”). Participants were given 4 min to solve each task. Trials ended when the child retrieved the sticker or when time was up. The experimenter never showed the correct solution of the task if children failed to retrieve the sticker. All children were rewarded with a sticker regardless of success. Total testing time was ~ 15 min.

## **Descriptive statistics by age groups**

Table S9. Number (and percentage) of valid trials in which ATU and Correct success were scored in the tasks used in Experiment 2, split by age groups.

| **Task type** | **Apparatus** | **Age group** (*n*_valid trials_) | **Associative tool use** | **Correct success** | **Incorrect success** |
| --- | --- | --- | --- | --- | --- |
| Tool set use | Box | 3y (10) | 0 (0%) | 0 (0%) | 0 (0%) |
|  |  | 4y (10) | 0 (0%) | 0 (0%) | 0 (0%) |
|  |  | 5y (6) | 2 (33.3%) | 1 (16.7%) | 0 (0%) |
|  |  | 6y (14) | 4 (28.6%) | 3 (21.4%) | 4 (28.6%) |
|  | Tube | 3y (10) | 1 (10.0%) | 0 (0%) | 0 (0%) |
|  |  | 4y (10) | 2 (20.0%) | 1 (10%) | 0 (0%) |
|  |  | 5y (6) | 1 (16.7%) | 1 (16.7%) | 1 (16.7%) |
|  |  | 6 (12) | 4 (33.3%) | 4 (33.3%) | 0 (0%) |
| Sequential tool use | Box | 3y (4) | 1 (25.0%) | 1 (25.0%) | 0 (0%) |
|  |  | 4y (15) | 2 (13.3%) | 2 (13.3%) | 1 (6.7%) |
|  |  | 5y (12) | 6 (50.0%) | 6 (50.0%) | 0 (0%) |
|  |  | 6y (7) | 2 (28.6%) | 2 (28.6%) | 0 (0%) |
|  | Tube | 3y (4) | 0 (0%) | 0 (0%) | 0 (0%) |
|  |  | 4y (15) | 0 (0%) | 0 (0%) | 0 (0%) |
|  |  | 5y (12) | 1 (8.3%) | 1 (8.3%) | 0 (0%) |
|  |  | 6y (8) | 1 (12.5%) | 1 (12.5%) | 0 (0%) |
| Multifunctional tool use | Box | 3y (6) | 1 (16.7%) | 1 (16.7%) | 0 (0%) |
|  |  | 4y (14) | 2 (14.3%) | 2 (14.3%) | 0 (0%) |
|  |  | 5y (9) | 3 (0.33%) | 3 (0.33%) | 0 (0%) |
|  |  | 6y (11) | 5 (45.4%) | 5 (45.4%) | 0 (0%) |
|  | Tube | 3y (6) | 2 (33.3%) | 1 (16.7%) | 0 (0%) |
|  |  | 4y (14) | 12 (85.7%) | 11 (78.6%) | 0 (0%) |
|  |  | 5y (9) | 8 (88.9%) | 8 (88.9%) | 0 (0%) |
|  |  | 6y (11) | 10 (90.9%) | 10 (90.9%) | 0 (0%) |

## **Description of performance in the three conditions (Tool set use, Sequential tool use, Multifunctional tool use), split by apparatus**

### **Tool set use**

In both versions of the Tool set use tasks, a vast majority of children picked up the stick tool and inserted it into the apparatus (Tables S10, S11). In the box version, many children failed to reach the next milestone (piercing the tin foil barrier), however (Fig. S6). This was mostly because of two reasons. One, some children managed to get the stick over the barrier and tried to reach the reward this way. This would have been unsuccessful as the stick would not bend down to reach the bucket nor would the bucket fit through the small slot the children managed to put the stick through. However, the stick got closer to the reward and this might have suggested to the children that they were using the correct strategy. Second, many children did try to pierce the tin foil barrier, but failed to do so, either because they did not seem to dare to break the foil or because they were pushing against the foil with too little force or too slowly.

The main hurdle in the tube version of the Toolset task seemed to be the removal of the cloth barrier after children inserted the stick tool (Fig. S7). First, many children accidentally dropped the stick into the apparatus and could not proceed with the task. Second, children often pushed the stick onto the cloth barrier and thus pushed it into the tube where the reward bucket was placed – possibly in an attempt to pierce the cloth barrier. Some children correctly tried to remove the barrier by pushing it to the side (not into the tube), but struggled with the physical aspects of this task.

Table S10. Descriptive statistics for the Tool set use version of the box apparatus of Experiment 2 (*n* = 40).

| Picked up stick (at any time) | Picked up stick as first tool | Perforated foil | Picked up pipecleaner (at any time) | Used pipecleaner to reach ball (ATU) | Correct success | Incorrect success |
| --- | --- | --- | --- | --- | --- | --- |
| 34 (85%) | 26 (65%) | 13 (32%) | 33 (82%) | 6 (15%) | 4 (10%) | 4 (10%) |

Table S11. Descriptive statistics for the Tool set use version of the tube apparatus of Experiment 2 (*n* = 38).

| Picked up stick (at any time) | Picked up stick as first tool | Removed cloth | Picked up rope (at any time) | Used rope to reach bucket (ATU) | Correct success | Incorrect success |
| --- | --- | --- | --- | --- | --- | --- |
| 34 (89%) | 6 (16%) | 13 (34%) | 34 (89%) | 8 (21%) | 6 (16%) | 1 (3%) |


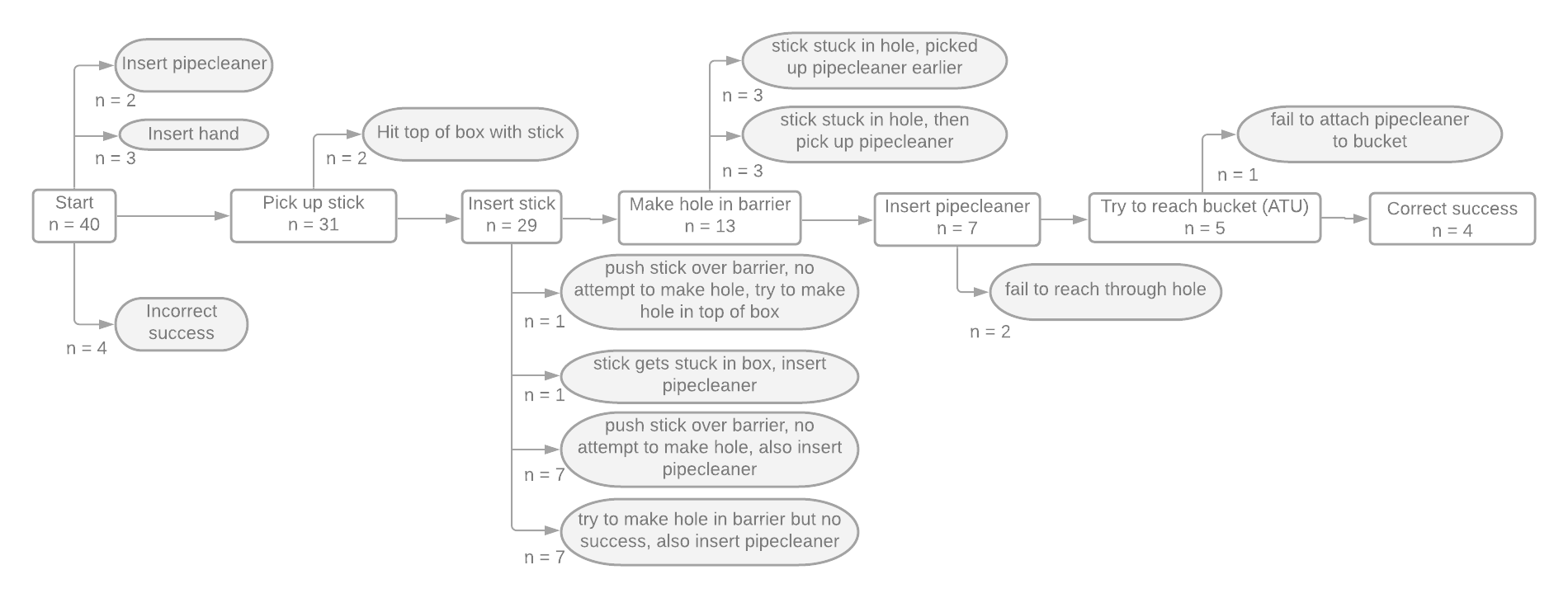


Fig. S8. Number of children at each milestone in the Toolset version of the box apparatus (Experiment 2), with grey fields depicting unsuccessful children. Incorrect success: Children retrieved bucket by inserting hand (1x) or by inserting pipecleaner and manoeuvring it over the barrier (3x).


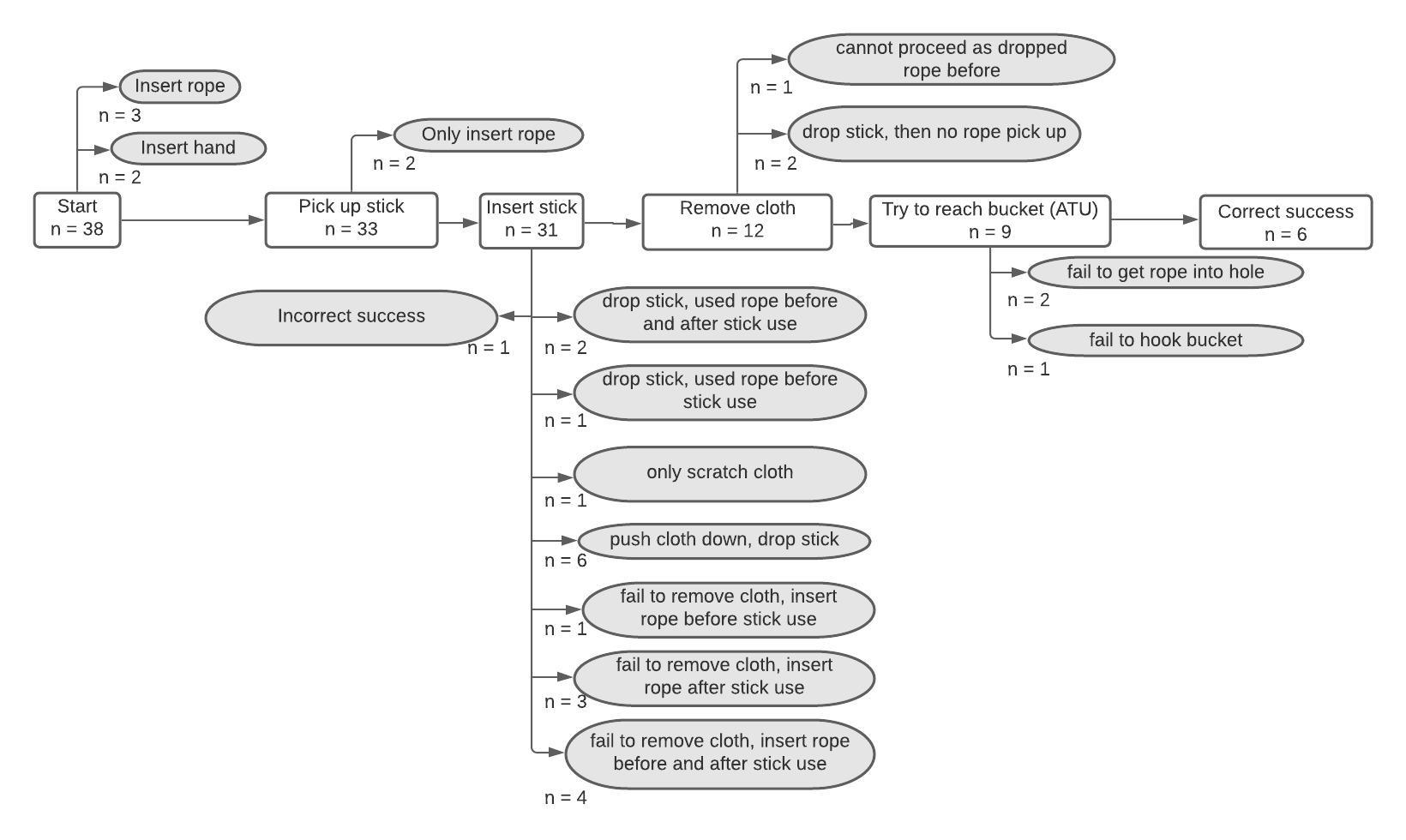


Fig. S9. Number of children at each milestone in the Toolset version of the tube apparatus (Experiment 2), with grey fields depicting unsuccessful children. Incorrect success: Child removed cloth by inserting hand.

### **Sequential tool use**

In both versions of the Sequential tool use tasks, a vast majority of children picked up the stick tool and inserted it into the apparatus (Tables S12, S13). However, only a small number of children retrieved the second tool from the apparatus. This was especially surprising in the box version, as the second tool was placed between the entrance of the box and the reward, and so was very often touched and moved by the stick when it was entered into the box (Fig. S8). In the box version, many of the children who put the stick into the box tried to reach the bucket and ignored the pipecleaner, even though the pipecleaner was “in the way” between them and the reward. In many cases children pushed the stick so far into the box in an attempt to reach the reward that they lost the stick and were not able to retrieve it.

In the tube version, noticing the second tool (i.e., the rope) inside the apparatus appeared to be more difficult than noticing the pipecleaner in the box version (Fig. S9). Many children were focused almost exclusively on the bucket and tried to reach it with the stick tool; often times they accidentally dropped the stick while trying to reach the bucket and were unable to proceed with the task. In sum, for both versions, it seemed that many children tried to reach the reward with the freely available stick tool and were unable to either recognize that this might not be the correct strategy or come up with a different idea.

As mentioned in the main manuscript, the box version yielded significantly higher rates of ATU (p = .027) and Correct success (p = .016) than the tube version. We can only speculate about possible reasons for this difference. For that, Figures S7 and S8 might be helpful, which show at which points in the solution process children failed or got “stuck”. In the Tube version, a large proportion of children (15/39 children – 38%) accidentally dropped the stick into the apparatus (no other causes of failure occurred that frequently). Dropping the stick implied the end of the task, so the child scored a failure in the ATU category, but this did not necessarily reflect an inability to show ATU. Therefore, one could speculate that if it had been possible for children to retrieve the stick once dropped (by a simple change to the task design), ATU rates might have been much higher in this Tube version, and reflect children’s tool-using abilities more realistically. In the box version of the Sequential tool use task, the most common way of failing the task was indeed also by getting the stick stuck in the apparatus. However, the difference to the other ways of failing the box task did not seem as pronounced as in the tube version.

Another reason could have been some children struggling to see the rope inside the tube apparatus. This could be a viable possibility because the rope was lying on a transparent part of the apparatus and so it was difficult to discern it from other parts of the box. In contrast, in the box version, the white pipecleaner was lying on a bright red floor of the apparatus and so the stark contrast made the pipecleaner visibly stick out.

Alternatively, however, when looking at the Sequential tool use tasks across Experiments 1 and 2 (see Table 2), one could also suggest that young children’s ATU rates in Sequential tool use tasks are generally low, and that the box version is deviating from this pattern. Again, one can only speculate about possible reasons for this deviation. One factor could be that in the box task, the second tool was lying directly “on the way” to the target. Children who were only fixated on the sticker and disregarded the pipecleaner would insert the stick to reach the bucket, and then inadvertently touch and move the pipecleaner lying in the box. This might have guided some children’s attention towards the pipecleaner and made them retrieve it, even though these children might not have immediately understood what the object could be used for. This could have been a facilitating factor in the box task which did not exist in the other Sequential tool use tasks. In Experiment 1, the second tools were placed in a different apparatus (which could be ignored completely) and in the tube task of Experiment 2, the rope was placed *next* to the hole leading down to the bottom of the tube, but not directly *across* the hole. Further research would be needed to test for these possibilities.

Table S12. Descriptive statistics for the Sequential tool use version of the box apparatus of Experiment 2 (*n* = 38).

| Picked up stick | Used stick to get pipecleaner | Obtained pipecleaner with stick | Used pipecleaner to get reward (ATU) | Correct success | Incorrect success |
| --- | --- | --- | --- | --- | --- |
| 36 (95%) | 18 (47%) | 13 (34%) | 11 (29%) | 11 (29%) | 1 (3%) |

Table S13. Descriptive statistics for the Sequential tool use version of the tube apparatus of Experiment 2 (*n* = 39).

| Picked up stick | Used stick to get rope | Obtained rope with stick | Used rope to get reward (ATU) | Correct success | Incorrect success |
| --- | --- | --- | --- | --- | --- |
| 38 (97%) | 6 (15%) | 2 (5%) | 2 (5%) | 2 (5%) | 0 (0%) |


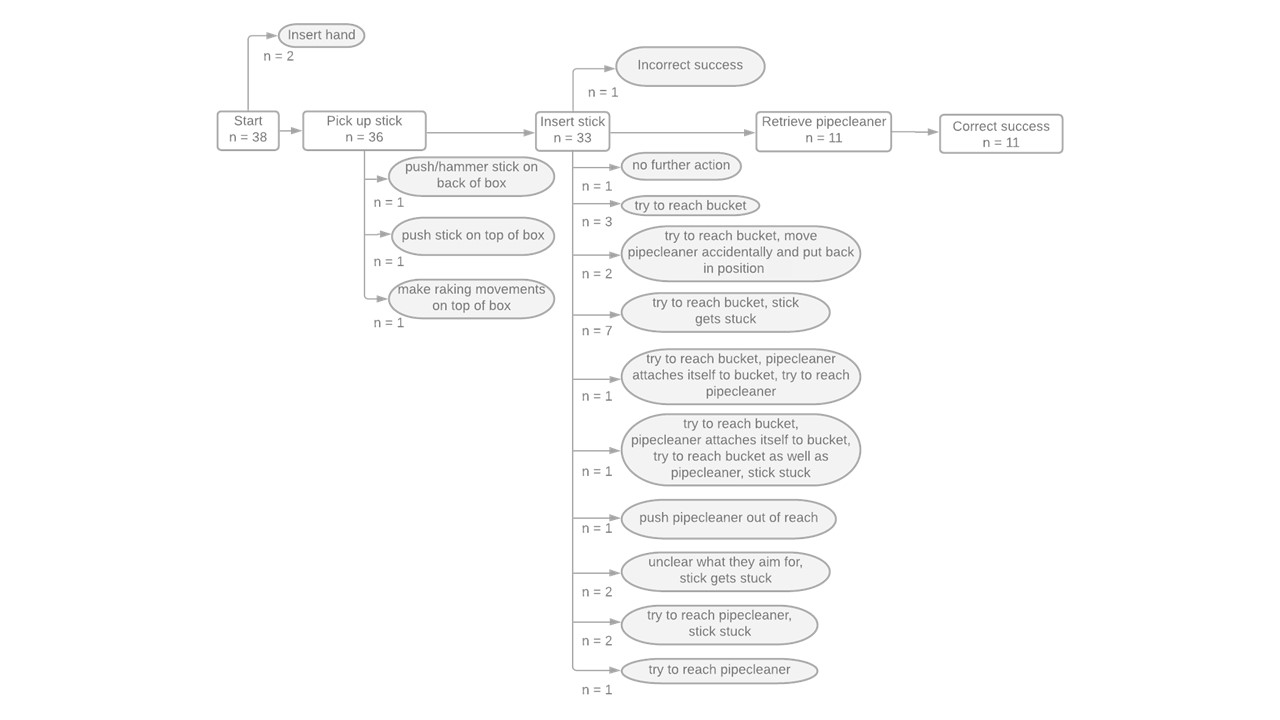


Fig. S10. Number of children at each milestone in the Sequential tool use version of the box apparatus (Experiment 2), with grey fields depicting unsuccessful children. Incorrect success: Child inserted stick and tried to get bucket, pipecleaner attached itself to the bucket, then child retrieved pipecleaner+bucket with the stick (no deliberate/purposeful ATU).


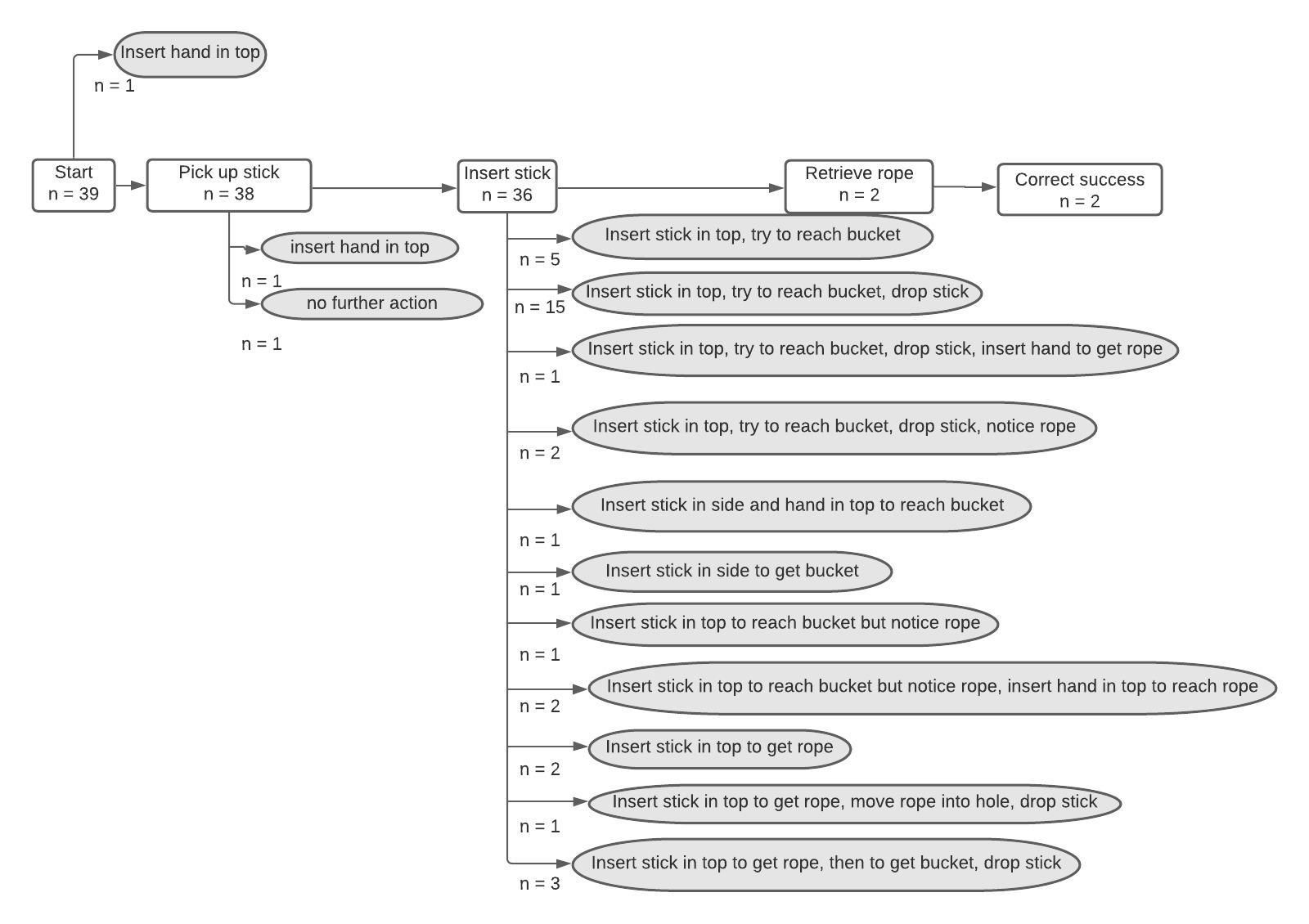


Fig. S11. Number of children at each milestone in the Sequential tool use version of the tube apparatus (Experiment 2), with grey fields depicting unsuccessful children.

### **Multifunctional tool use**

In both versions of the Multifunctional tool use tasks, a vast majority of children picked up the tool and inserted it into the apparatus (Tables S14, S15). Success rates in the tube version were higher than in the box version. In fact, the tube version of the Multifunctional tool use condition was the task with the highest success rates overall. In comparison to the box version of the Tool set use condition, the two main hurdles were removed/reduced in the Multifunctional tool use condition: it was impossible to drop the stick into the apparatus due to its length, and also due to the length of the tool getting access to the reward by moving the cloth seemed easier, even if children simply pushed the cloth into the tube (Fig. S11). In the box version, similar to the box version of the Toolset condition, many children did not perforate the foil, either because they used too little force or because they put the tool over the barrier and got stuck with this attempt (Fig. S10).

Table S14. Descriptive statistics for the Multifunctional tool use version of the box apparatus of Experiment 2 (*n* = 40).

| Picked up tool (at any time) | Aimed to perforate foil with tool | Perforated foil with tool | Used tool to reach ball after perforating | Correct success | Incorrect success |
| --- | --- | --- | --- | --- | --- |
| 38 (95%) | 22 (55%) | 11 (27%) | 11 (27%) | 11 (27%) | 0 (0%) |

Table S15. Descriptive statistics for the Tool set use version of the tube apparatus of Experiment 2 (*n* = 40).

| Picked up tool (at any time) | Tried to remove cloth with tool | Removed cloth | Try to reach bucket with tool | Correct success | Incorrect success |
| --- | --- | --- | --- | --- | --- |
| 37 (92%) | 32 (80%) | 32 (80%) | 32 (80%) | 30 (75%) | 0 (0%) |


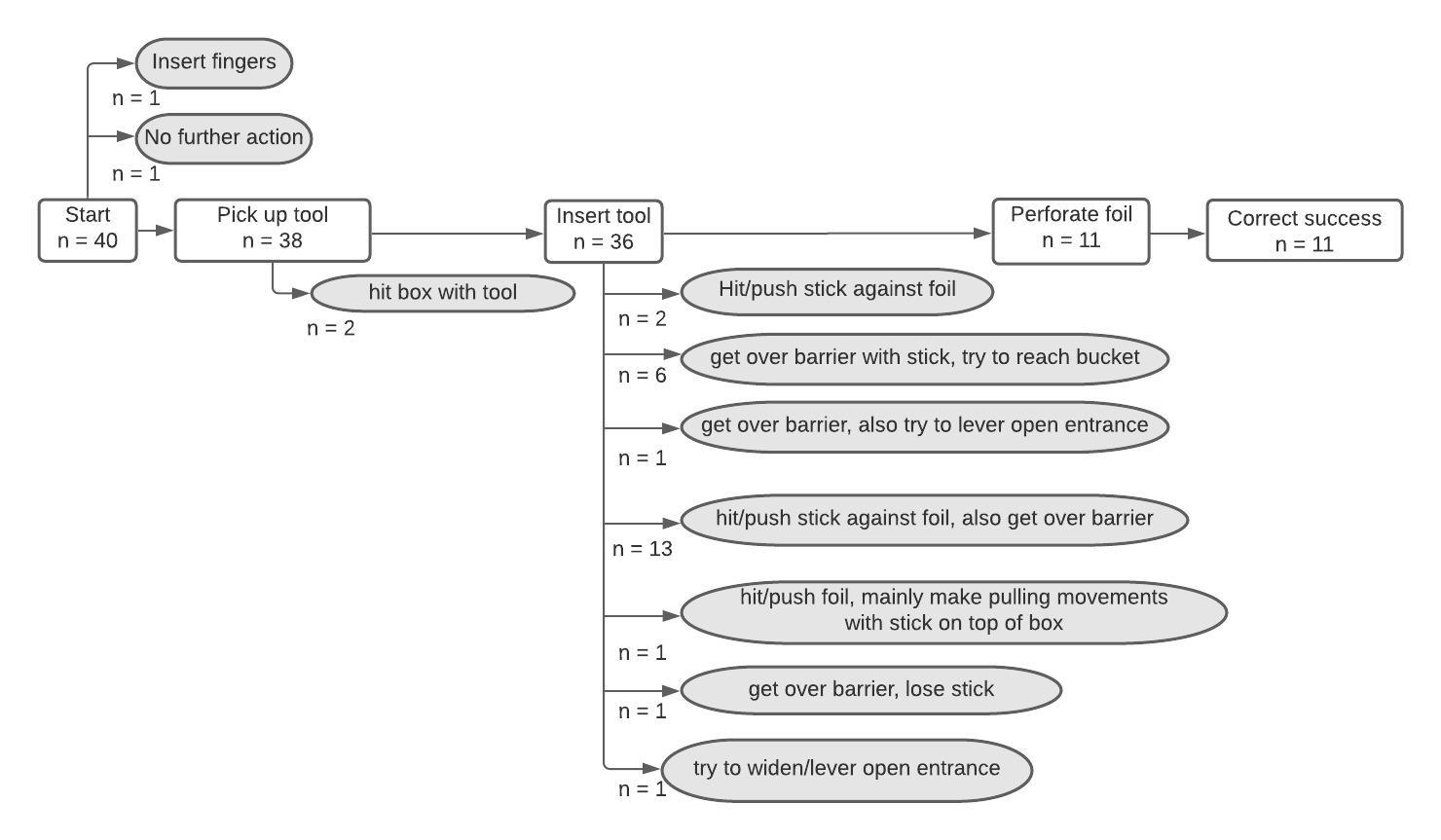


Fig. S12. Number of children at each milestone in the Multifunctional tool use version of the box apparatus (Experiment 2), with grey fields depicting unsuccessful children.


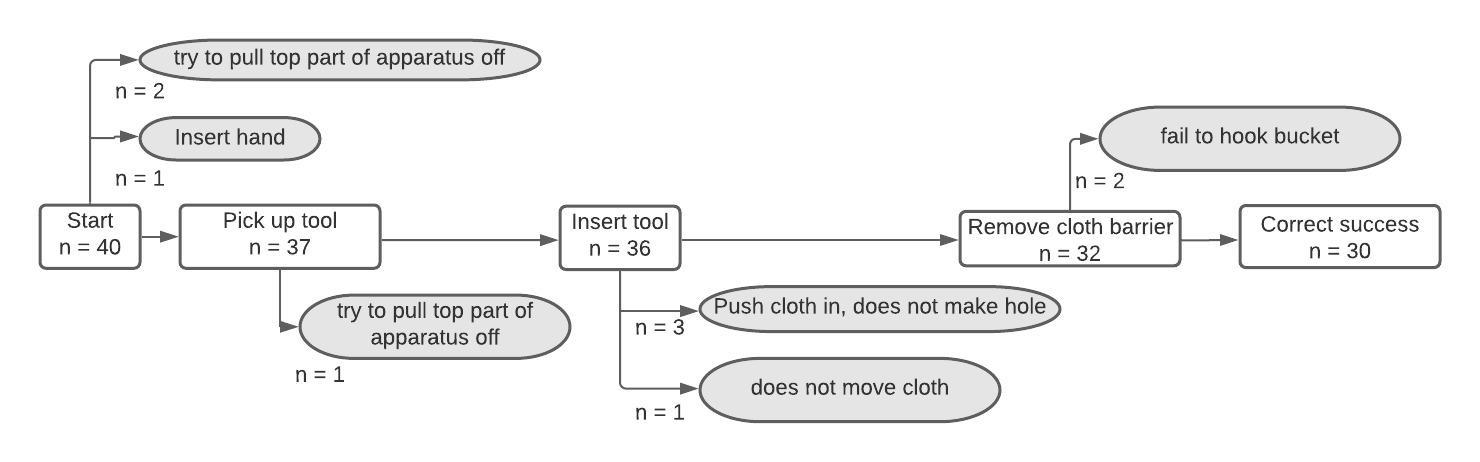


Fig. S13. Number of children at each milestone in the Multifunctional tool use version of the tube apparatus (Experiment 2), with grey fields depicting unsuccessful children.

## **Results from GLMMs**

Table S16. Results of the model for Experiment 2 with ATU as dependent variable (estimates, together with standard errors, bootstrapped confidence limits, significance tests and minimum and maximum of model estimates when excluding individuals one at a time, odds ratios).

| **Term** | **Estimate** | **SE** | **Lower CI** | **Upper CI** | **p** | **min** | **max** | **SD** | **Odds ratio [95% CI]** |
| --- | --- | --- | --- | --- | --- | --- | --- | --- | --- |
| Intercept^1^ | 0.164 | 0.235 | -0.291 | 0.680 | ^2^ | 0.098 | 0.249 |  |  |
| Age (in months, z-transformed) | 0.616 | 0.167 | 0.317 | 1.018 | **< .001** | 0.581 | 0.668 |  | 1.85 [1.37; 2.77] |
| Condition: Sequential vs Multifunctional^3^ | -1.835 | 0.393 | -2.963 | -1.086 | **< .001** | -1.978 | -1.776 |  | 0.16 [0.05; 0.34] |
| Condition: Tool set vs Multifunctional tool use^3^ | -1.808 | 0.391 | -2.824 | -1.129 | **< .001** | -1.916 | -1.754 |  | 0.16 [0.06; 0.32] |
| Condition: Tool set vs Sequential tool use^3^ | 0.026 | 0.435 | -0.891 | 0.933 | = .951 | -0.062 | 0.185 |  | ^4^ |
| Random intercept for ID |  |  |  |  |  |  |  | 0.000 |  |

Notes.^1^The numbers for the intercept are those retrieved with Metatool use as the reference category. ^2^ Not shown due to limited interpretability. ^3^The overall effect of condition was χ^2^ (2) = 33.98, *p* < .001. ^4^Not shown as not significant.

Table S17. Results of the model for Experiment 2 with Correct success as dependent variable (estimates, together with standard errors, bootstrapped confidence limits, significance tests and minimum and maximum of model estimates when excluding individuals one at a time, odds ratios).

| **Term** | **Estimate** | **SE** | **Lower CI** | **Upper CI** | **p** | **min** | **max** | **SD** | **Odds ratio [95% CI]** |
| --- | --- | --- | --- | --- | --- | --- | --- | --- | --- |
| Intercept^1^ | 0.056 | 0.241 | -0.424 | 0.551 | ^2^ | -0.022 | 0.147 |  |  |
| Age (in months, z-transformed) | 0.787 | 0.183 | 0.461 | 1.256 | **< .001** | 0.751 | 0.849 |  | 2.20 [1.58; 3.51] |
| Condition: Sequential vs Multifunctional^3^ | -1.782 | 0.401 | -2.771 | -1.109 | **< .001** | -1.920 | -1.718 |  | 0.17 [0.06; 0.33]) |
| Condition: Tool set vs Multifunctional tool use^3^ | -2.210 | 0.439 | -3.483 | -1.465 | **< .001** | -2.339 | -2.149 |  | 0.11 [0.03; 0.23] |
| Condition: Tool set vs Sequential tool use^3^ | -0.428 | 0.472 | -1.435 | 0.533 | = .365 | -0.544 | -0.268 |  | ^4^ |
| Random intercept for ID |  |  |  |  |  |  |  | 0.000 |  |

Notes.^1^ The numbers for the intercept are those retrieved with Metatool use as the reference category. ^2^ Not shown due to limited interpretability. ^3^The overall effect of condition was χ^2^ (3) = 55.51, *p* < .001. ^4^Not shown as not significant.
